# Identification of New Hematopoietic Cell Subsets with a Polyclonal Antibody Library Specific for Neglected Proteins

**DOI:** 10.1371/journal.pone.0034395

**Published:** 2012-04-04

**Authors:** Monica Moro, Mariacristina Crosti, Pasquale Creo, Pierangela Gallina, Serena Curti, Elisa Sugliano, Rossana Scavelli, Davide Cattaneo, Elena Canidio, Maurizio Marconi, Paolo Rebulla, Paolo Sarmientos, Giuseppe Viale, Massimiliano Pagani, Sergio Abrignani

**Affiliations:** 1 Fondazione Istituto Nazionale di Genetica Molecolare, INGM, Milan, Italy; 2 PRIMM, Milan, Italy; 3 IRCCS Ca' Granda Ospedale Maggiore Policlinico, Milan, Italy; 4 European Institute of Oncology, IEO, Milan, Italy; Emory University, United States of America

## Abstract

The identification of new markers, the expression of which defines new phenotipically and functionally distinct cell subsets, is a main objective in cell biology. We have addressed the issue of identifying new cell specific markers with a reverse proteomic approach whereby approximately 1700 human open reading frames encoding proteins predicted to be transmembrane or secreted have been selected in silico for being poorly known, cloned and expressed in bacteria. These proteins have been purified and used to immunize mice with the aim of obtaining polyclonal antisera mostly specific for linear epitopes. Such a library, made of about 1600 different polyclonal antisera, has been obtained and screened by flow cytometry on cord blood derived CD34+CD45dim cells and on peripheral blood derived mature lymphocytes (PBLs). We identified three new proteins expressed by fractions of CD34+CD45dim cells and eight new proteins expressed by fractions of PBLs. Remarkably, we identified proteins the presence of which had not been demonstrated previously by transcriptomic analysis. From the functional point of view, looking at new proteins expressed on CD34+CD45dim cells, we identified one cell surface protein (MOSC-1) the expression of which on a minority of CD34+ progenitors marks those CD34+CD45dim cells that will go toward monocyte/granulocyte differentiation. In conclusion, we show a new way of looking at the membranome by assessing expression of generally neglected proteins with a library of polyclonal antisera, and in so doing we have identified new potential subsets of hematopoietic progenitors and of mature PBLs.

## Introduction

The identification of phenotypically distinct cell subsets within apparently homogeneous cell populations is a key step toward the identification and functional characterization of new cell subsets having both specific effector functions and differentiation pathways. Immunological studies offer one of the best examples of this assumption. From the discovery of the main T lymphocyte subsets in the 1970s [1] to the recent identification of the poorly represented regulatory subsets such as Treg and Th17 [2], [3], [4], [5], every time that a new T cell subset has been characterized phenotypically, a significant improvement in the understandings of the effector functions of the immune system has been subsequently achieved.

All the human genome has been sequenced and annotated [6], [7], [8], [9], and a significant amount of gene products have been studied in some details. However, the distribution and function of a large fraction of human gene products is still unknown [6], [10], [11], [12]. Generally, in the present post-genomic era, the identification of new proteins on cells of interest has resulted either from classical proteomics approaches [13], [14], [15] or from gene expression profile analyses [16], [17]. Both these approaches are sensitive enough to identify new genes and proteins expressed in a given cell population [18], [19], [20], [21]. However, it is impossible to assess whether differences in the expression levels of proteins occur in all of the cells analyzed or in a subset of them. It is therefore difficult to study those cell subsets or lineages that are poorly represented within a population and the amount of starting material may remarkably affect the results obtained with these methods [22].

One of the most effective ways to identify and characterize new proteins is the use of specific antibodies. We therefore developed an experimental approach aimed at obtaining a polyclonal antibody library composed of individual antisera specific for most of those thousands of poorly characterized human proteins located outside the cell. We focused our attention on proteins that are predicted *in silico* to be secreted [23] or transmembrane, and to have at least one domain predicted to be “outside” the cell. These proteins are therefore likely to be used by cells to interact with the external milieu. We assumed that it would have been possible to characterize unknown gene products as new subset-defining proteins with specific antibodies.

We previously selected *in silico* 5086 ORFs potentially encoding for membrane or secreted proteins so far poorly characterized in distribution and function. These genes have been cloned and expressed in *Escherichia coli*. The recombinant proteins have been purified and used to immunize groups of five mice generating a library of 1559 polyclonal antisera (Data S1, [24])

In this work we describe the use of this library to assess the expression of poorly characterized membrane bound proteins on immature or mature hematopoietic cells from healthy donors by flow cytometry. These analyses were performed on cord-blood derived CD34+CD45dim cells or on Peripheral Blood Lymphocytes (PBLs) and resulted in the identification of eight new proteins expressed by PBLs subset and of three new proteins expressed on subsets of CD34+CD45dim cells.

We show that this high throughput screening is suitable for the study of very poorly represented cell populations, such as CD34+ cell subsets within the whole cord blood cell population. Moreover, the use of flow cytometry allows not only to estimate the percentage of cells expressing a given cell surface protein but also to separate live positive cells for further studying phenotypical and functional features of the newly identified population.

## Methods

### Preparation of a library of polyclonal antisera specific for human unknown proteins

Details on the library of antisera used in the present work are presented in full in Data S1. Briefly the genes coding for proteins predicted to be transmembrane or secreted with unknown distribution and functions were selected and expressed in *E. coli*. The recombinant proteins were then purified by Ion Metal Affinity Chromatography (IMAC) and used to immunize groups of five mice to produce polyclonal antisera.

### Cell Preparation and Purification

Peripheral blood and cord blood were obtained from healthy donors after the signature of specific informed consent, fulfilling the requirement of the ethical committee of the Fondazione IRCCS Ca' Granda Ospedale Maggiore Policlinico. The mononuclear cells from healthy donors blood and cord blood were obtained by density gradient centrifugation on Lympholyte-H (Cedarlane Laboratories Ltd) and immediately analyzed after the separation. PBL activation was induced on overnight by the addition on medium phytohemagglutinin (PHA) 1 µg/ml (ROCHE Diagnostic GmbH) and IL-2 100 U/ml (Novartis). The cells were cultured at 37°C and 5% CO_2_ in RPMI supplemented with 10%FBS (EuroClone S.p.A) and antibiotics (GIBCO)

### Flow Cytometry

Flow cytometry screening of the library was performed on 2 to 5×10^5^ PBL or 5×10^6^ cord blood mononuclear cells. Resting or activated PBMCs were stained in a three-step procedure. Cells were incubated for 20 min at room temperature with 50% NHS (Euroclone) in PBS (Euroclone) to block Fc receptor; then incubated with anti-serum at the optimal concentration (1∶50 to 1∶450 dilution) in 5%NHS-PBS (Facs wash) for 10 min at 4°C, washing two time at 1500 rpm for 3 min and finally incubated with Goat anti mouse RPE (SouthernBiotech) at 1∶200 dilution for 10 min at 4°C, washing two time with Facs wash at 1500 rpm for 3 min.

Multi color FACS analysis on cord blood cells was performed as following. Cells were stained for the first three steps as PBL then incubated with mouse IgG (SIGMA) at 4 mg/10^6^ cells for 1 hour at 4°C then the following mAbs were added: CD34, (IOT Coulter), CD45 (ImmunoTools), or for PBL in multi color CD3, CD19, CD56, CD71, Glycophorin A, CD7, CD33, CD38, CD10 (BD Biosciences), CD117 (IOT Coulter), CD133/2 (Macs Miltenyi Biotec GmbH), Mouse isotype-matched was used as negative controls. The samples were acquired using a FACScanto II analyzer (Becton Dickinson) and data were processed with the program FlowJo (Flow Cytometry Analysis Software).

### Amplification by RT PCR of the transcripts corresponding to the newly identified proteins

RNA extraction was performed using RNeasy Mini Kit or RNeasy Micro Kit (QIAGEN) on peripheral blood, cord blood cells or magnetically purified CD34 positive cells using CD34 Miltenyi microbeads kit. Cells were then lysates and homogenized with QIAshredder homogenizer (QIAGEN).

cDNA synthesis was performed using SuperScript III First-Strand Synthesis SuperMix (Invitrogen).

RT-PCR primers 5′-3′ sequences:

TMCC1 379 fw CAGGAGGAGCGATATAGATGTG

  379 rev TGGCTACAGTGGAGACAAAG

MOSC-1 194 fw TTCCTGAAGTCACAGCCCTAC

  194 rev GCATCTGGAACAAGCCATCAC

SUSD3 452 fw A TTGTGAGCTGTGCCATCATCC

  452 rev A TGTGGTGAAGCTGTGGTTGTC

TMEM126 314 fw GGCGACATTTGGAACAAC

  314 rev TTTGGTGGCAGTGGAACG

LPPR2 1174 fw AGCGATGTACGTGACTCTC

  1174 rev CAGTTCTGCGACTTGGATG

GSG1-L 444 fw b TCTGTCACCACGCTCAACTCC

  444 rev b AAGACCCAGCACTGTCGGTTC

TMEM38 147 fw CACCCAGCATCTGGCAATATC

  147 rev GCAACATCTACCGGCTTTGAG

KRTCAP3 665 fw AGGACTGCTGGATCCTCTG

  665 rev GCACCTGCTGTCCTAAACC

CRISP-1 26nested2 fw TAAGCTCGTCACCGACTTG

  26nested2 fw CTCCTCATCGTCACAGCATAG

  26a fw ACACAACGCCCTCAGGAGAAG

  26a rev TGGCGGCAAGATGCAATGG

  26b fw GTTTGGGCCACATCTTAC

  26b rev CGTCACAGCATAGAACAG

### Clonogenic Assay

Clonogenic assays were performed using αserum positive cells, isolated using FACSAria cell sorter (Becton Dikinson). The cells were plated at 250 cells/plate in METHOCULT H4433 complete methylcellulose medium (StemCell Technologies). Cultures were incubated for 14 days in incubator adjusted to 37°C, 5%CO_2_ and >95% humidity.

### Transient Transfection

To carry out transient transfection experiments, we used MicroPorator MP-100, a pipette-type electroporation system (NanoEnTek Inc). The cells were dissociated by a brief treatment with trypsin-EDTA (Euroclone). The indicated plasmid DNAs were introduced into 5×10^5^ dissociated cells in 10 µl volumes according to manufacturer's instructions (3 pulses with 10 msec duration at 1600 voltage; Digital Bio Technology).

Electroporated cells were then seeded into 6-well culture dishes (Nunc) containing 2 ml of culture media. After 24–48 hrs cells were harvested and lysated for Western blot analysis

### Western blot analysis

Whole-cell extracts were loaded on pre-casted sodium dodecyl sulfate (SDS)-polyacrylamide gels (Bio-Rad Laboratories S.r.l.) and transferred to Hybond-P PVDF membranes (GE Healthcare) using Towbin's buffer (25 mM Tris, pH 8.3, 192 mM glycine and 20% methanol). The blots were blocked in PBS containing 0.5% Tween 20 (SIGMA) and 5% not fat milk and incubated with primary antibody (anti-myc mAb 9E10 clone or antiserum) at room temperature for 1 hr. The blots were then washed four times with PBS/0.5% Tween-20. Primary antibody binding was subsequently detected by incubation for 1 hour with secondary antibodies goat anti mouse HRP (SouthernBiotech). The blots were then developed using the SuperSignal West Dura Extended Duration Substrate (Thermo Fisher Scientific).

## Results

### Preparation and validation of a library of polyclonal antisera specific for human unknown proteins

**Figure 1:**
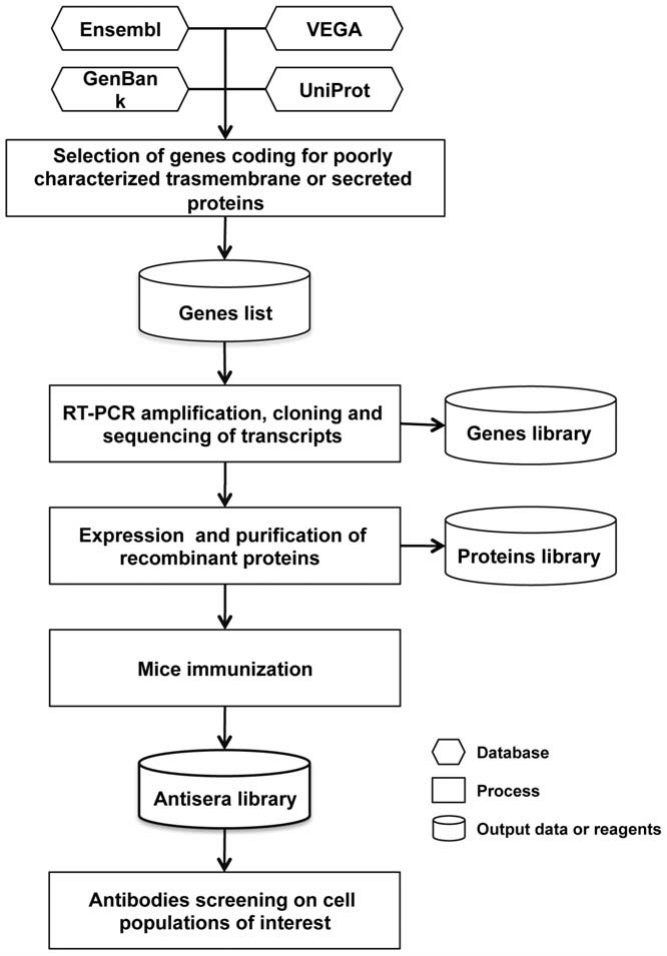
Schematic representation of the antisera library generation.

We identified in the human genome 7902 genes coding for proteins that are predicted to be either transmembrane or secreted. For about two-thirds of them there is a little information on distribution and function. In order to gain insight into the function of these neglected proteins we generated a specific antibody library against proteins poorly characterized predicted as integral membrane or secreted. We have selected about 3000 genes, cloned and expressed them in *E. coli*, purified the recombinant proteins and immunized groups of five mice with each individual protein (Figure 1). We thus obtained a library of 1559 mouse antisera specific for 1287 poorly known human secreted or transmembrane proteins (for 214 genes more than one protein fragment were selected) (Griffantini, Pagani et al., Data S1).

As our mice antisera were raised against human proteins expressed in bacteria, most likely they are directed against linear epitopes, so a key question was whether such antibodies would recognize the corresponding proteins on the surface of human cells. To obtain proof of concept that our antibody library had the potential to identify new cell surface proteins, we produced in bacteria, with the same technology described above, antisera specific for twenty well characterized proteins (*i.e.*, with assigned CD numbers) known to be present on hematopoietic cells and for which very good monoclonal antibodies exist (Table 1). We then asked whether we would be able to “identify” these proteins on living cells by flow cytometry or on fixed and embedded tissues by immuno-hystochemistry (IHC). Table 1 summarizes the whole results and shows that by flow cytometry we could “identify” 12/20 (60%) well-characterized proteins on the surface of living PBLs. Remarkably, with IHC on fixed and embedded lymph node tissues we “identified” 11/14 (85%) known proteins in lymphoid cells.

**Table 1:** Comparison of the results obtained with antisera specific for well-known proteins assessed both by FACS on PBMCs from healthy donors and by IHC on inflamed lymph nodes.

*Symbol*	*FACS*	*IHC*
CD1d	positive	nd
CD2	positive	positive
CD3d	negative	negative
CD3e	negative	positive
CD8a	positive	positive
CD8b	negative	positive
CD27	positive	positive
CD33	positive	nd
CD38	positive	nd
CD45	negative	positive
CD72	positive	nd
CD122	negative	nd
CD161	negative	nd
CD25, IL-2Ra	positive	positive
CD69	negative	positive
CD71	negative	positive
CD80	positive	negative
CD86	positive	positive
CD34	negative	nd
CD133	positive	nd

**Figure 2:**
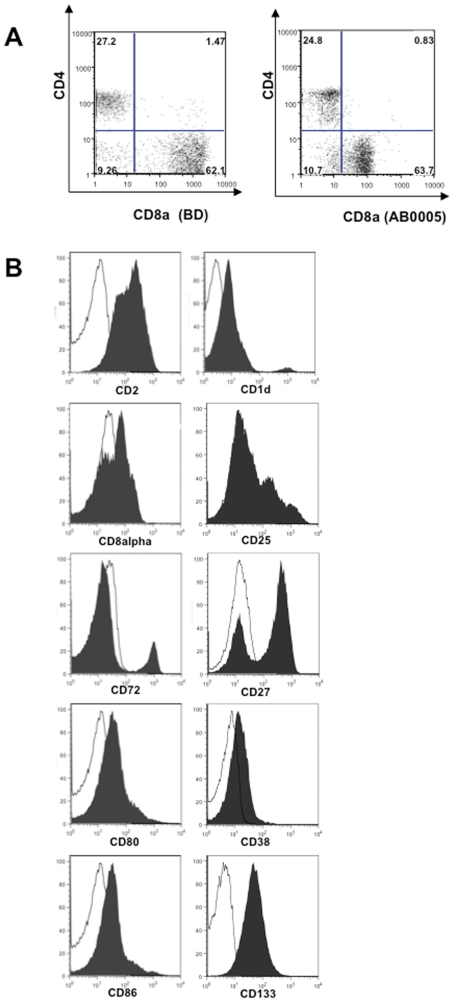
FACS analysis with sera specific for well-known proteins. (A) Comparison of the CD8 staining performed on PBL with either a commercially available anti CD8 mAb (BD biosciences) or the anti CD8 alpha antiserum at 1∶100 dilution points. Both the samples were stained also with commercially available anti CD3 and anti CD4 mAb (BD biosciences). The distribution of CD4 and CD8 is analyzed upon gating on CD3 positive cells. (B) Examples of staining with antisera from the library. PBLs from healthy donors were stained with anti CD2, CD1d, CD8 alpha, CD25, CD72, CD80, CD38, and CD86. The expression of CD25 was assessed upon a 24 hours activation of PBLs with 1 µg/ml of PHA. The expression of CD80 and CD86 was assessed upon gating on monocytes after a 24 hours activation of PBLs with 1 µg/ml of PHA. The expression of CD133 was analyzed on cord blood derived CD34+, CD45dim cells. Serum from not immunized mice was used as negative control in all the stainings.

Figure 2 shows representative stainings of lymphocytes with these antisera by flow cytometry. PBLs were stained with our polyclonal anti-CD8 antiserum in comparison with a commercial anti-CD8 monoclonal antibody (mAb) used alone or in a combination with commercial anti-CD3 and anti-CD4 mAbs (Panel A). Other examples of staining with antisera from the library are shown in Panel B. PBMCs from healthy donors were stained with anti CD2, CD1d, CD8 alpha, CD25, CD72, CD80, CD38, CD86. The expression of CD25 was assessed upon a 24 hours activation of PBMCs with 1 µg/ml of PHA. The expression of CD80 and CD86 was assessed upon gating on monocytes after a 24 hours activation of PBLs with 1 µg/ml of PHA. The expression of CD133 was analyzed on cord blood derived CD34+, CD45dim cells. In all the cases serum from not immunized mice was used as negative control. For all the proteins analyzed the staining was consistent with the expected percentage of positive cells. The antisera specific for well-known proteins were tested also on inflamed lymph-nodes tissues by IHC. The representative IHC experiments on lymph nodes are shown in Figure S1.

From the above results we concluded that our approach would be suitable to identify new molecules on a cell population of choice by IHC or flow cytometry, and most importantly, that antisera from the library can be used in multi-parametric analysis by flow cytometry. Although the use of IHC would result in a lower number of false negative antibodies compared to flow cytometry, we decided to utilize flow cytometry to screen human hematopoietic cells, because of the possibility to gate and analyze very rare cells subsets (<1–2%) which would pass mostly undetected in a screening performed by IHC.

### Identification of new proteins expressed on the surface of PBLs and CD34+CD45dim cells

After validating the approach, we screened our antisera library on resting or activated PBLs and cord blood samples with the aim at identifying molecules able to define new cell subsets within PBLs and CD34+CD45dim cells. Each individual antiserum was tested in three dilution points on at least three independent PBLs and at least three independent cord blood samples. On PBLs, the screening was performed on 5×10^5^ cells from either resting or phytohemagglutinin (PHA)-stimulated lymphocytes. On cord blood samples, 5×10^6^ cells were analyzed to eventually gate on 1000–2000 CD34+, CD45dim cells. In the search for new cell subsets, we concentrated our efforts on antisera that were positive for a fraction of the population we were interested in. Antisera that resulted positive with these criteria after the first screening on PBLs or CD34+ cells were validated on cell samples from ten additional independent donors. Finally, to confirm the presence of the transcript corresponding to the protein recognized by the antiserum we assessed mRNAs by RT-PCR analysis on purified cells.

**Figure 3:**
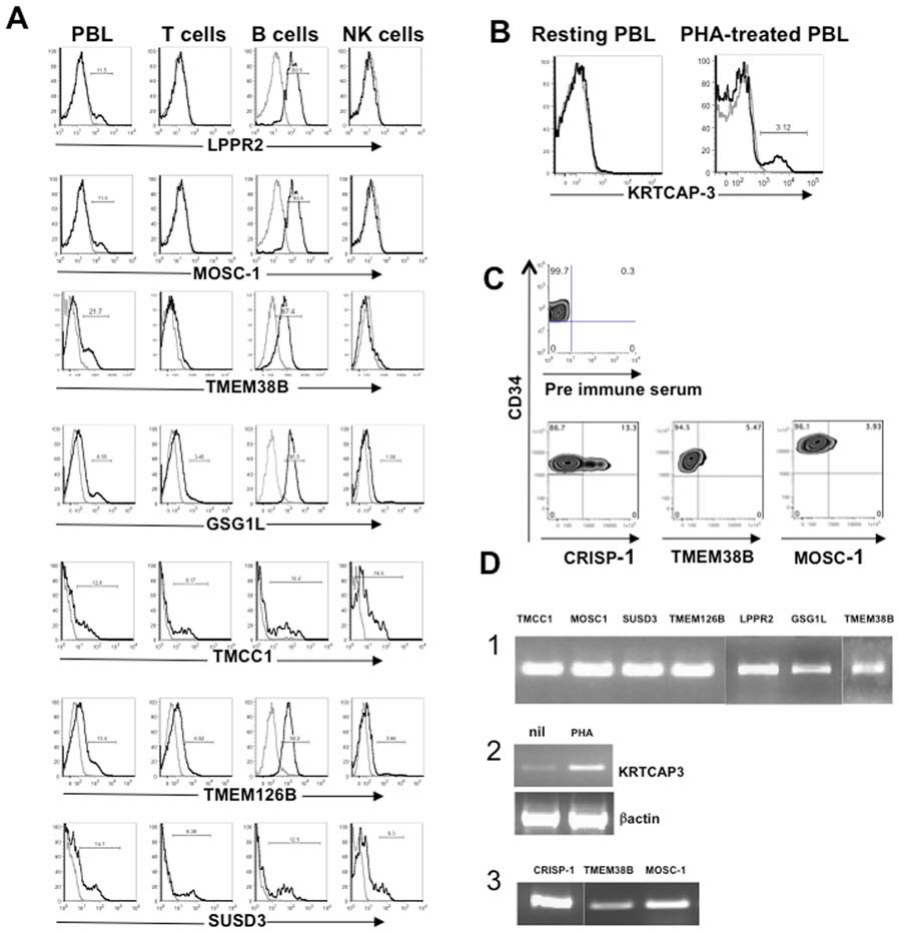
Results of sera screening by FACS on PBL and Cord Blood cells. (A) FACS analysis of sera positive on PBLs. PBLs were stained with the indicated sera at the optimal dilution point (1∶50 to 1∶200). The samples were stained also with anti CD3, anti CD19 and anti CD56 mAbs to analyze the sera reactivity upon gating on the different subpopulations. A plot representative of five different donors is shown for each serum. (B) KRTCAP-3 specific serum recognizes PHA-treated cells. PBMCs are treated for 24 hours with 1 µg/ml of PHA. After the treatment both un-stimulated and treated cells are stained with the KRTCAP-3-specific serum. (C) FACS analysis of sera positive on cord blood cells. Cord blood mononuclear cells are stained with the indicated sera at the optimal concentration (1∶50 to 1∶100). The samples are stained also with anti CD45 and anti CD34 mAbs to perform the analysis upon gating on CD34highCD45dim. A plot representative of a least 3 independent donors is shown. Il all the cases (A,B,C,) a staining with the serum of not immunized mice was used as negative control. (D) RT-PCR analysis. a- cDNA from total PBMC were amplified with primers specific for the indicated proteins. b- cDNA from both un-stimulated and PHA-treated PBMC was amplified with KRTCAP-3 specific primers. KRTCAP3 expression is up regulated two to three times. Beta actin amplification is used as normalization. c- cDNA samples from CD34+CD45dim cells were generated by retro-transcription of RNA extracted from a pool of CD34 positive cells from 2–3 independent cord blood units magnetically purified using the Miltenyi CD34 microbeads kit according the manufacturer instruction. The purity of the CD34+CD45dim cells was usually >99%. The samples were amplified with primers specific for the indicated proteins and described in the Methods section.

Figure 3 shows that the high throughput screening of hematopoietic cells with 1559 antisera led to the identification of seven molecules expressed on PBL subsets (panel A), one new molecule up-regulated on a subset of activated PBLs (Panel B) and three new molecules expressed on a subset of CD34+CD45dim cells (Panel C). To characterize the newly identified cell subsets, we performed a multicolor FACS analysis of the antisera in combination with monoclonal antibodies specific for the known main subsets of PBLs (CD3+ T lymphocytes, CD19+ B lymphocytes, or CD56+ Natural Killer cells) or CD34+ cells. Figure 3A shows that four out of seven new molecules we identified on PBLs (i.e., LPPR2, MOSC-1, TMEM38B and GSG1L) are mainly present on B lymphocytes, whereas the other three antisera specific for TMCC1, TMEM126B and SUSD3 are positive on both T and B lymphocyte subsets. Figure S2 shows the validation of this analysis on ten additional donors. The presence of the transcripts was confirmed by RT-PCR performed either on total peripheral blood mononuclear cells (PBMCs) (Figure 3D, panel 1 and 2) or on CD34+CD45dim cells purified magnetically with an anti-CD34 mAb out of cord blood cells (Figure 3D, panel 3).

**Figure 4:**
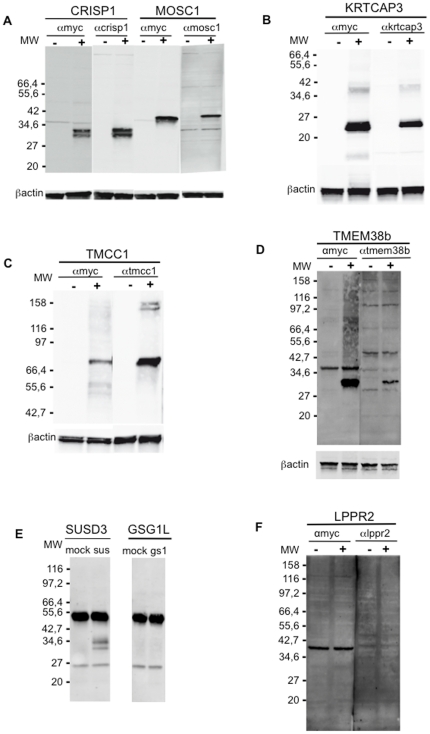
Assessment of antisera specificity on Hela transfected cells. Hela cells were transiently transfected with a myc-tag version of the proteins identified with the sera library. At 24 hours from the transfection cells were lysated as described in the Method section. 40 µg of total proteins were loaded on SDS page and a WB analysis was performed using both an anti myc mAb (9E10 clone) and the corresponding antiserum. (A) WB analysis of Hela cells transfected with CRISP-1 and MOSC-1. In both the cases the anti myc mAb and the specific antiserum recognized a protein of the expected molecular weight that is not present in the cells transfected with the mock vector. A comparable result was obtained wit KRTCAP-3 (B), TMCC-1 (C), TMEM38B (D) and SUSD3 (E) transfected cells. The WB analysis of GSG1-L cells (E) and LPPR2 cells (F) shows that neither the anti myc nor the specific antiserum is able to recognize in a specific way a protein in transfected cells.

Since the antisera of our library were raised in mice by using proteins produced in bacteria, the possibility exists that they are cross-reactive on human cells ex vivo. To rule out the possibility of sera cross-reaction toward unrelated molecules, we cloned the genes coding for the putatively identified proteins in a mammalian expression vector in frame with a c-myc tag, transiently transfected HeLa cells and assessed protein expression by western blot using either the specific antiserum or an anti c-myc mAb. Figure 4 shows that antisera recognized a band of the expected molecular weight, whereas only two antisera (putatively specific for LPPR2 and GSG1L) did not recognize a band of the expected molecular weight indicating these two antisera could be cross-reactive, originating false positive signals in the screening despite the positive RT-PCR. These results were confirmed by FACS analysis with the specific antisera on the same transfectants as shown in Figure S3, indicating that a validation of the data coming from the primary screening either by FACS or Western Blot is mandatory to further exclude false positive signals.

From all the above experiments, we conclude that our reverse proteomic approach to analyze the membranome is specific and sensitive enough to allow the identification of new hematopoietic cell subsets. Notably, we identified proteins the presence of which had not been demonstrated previously by transcriptomic analysis. For instance, we found (Fig. 3C) that a fraction of CD34+CD45dim cells express MOSC-1 on their surface, whereas previous gene expression profile analyses indicated a possible expression of MOSC-1 on peripheral blood monocytes, myeloid hematopoietic precursors but not on more immature CD34+CD45dim cells (http://symatlas.gnf.org/SymAtlas).

### Identification of MOSC-1 as a marker of mono-granulocyte development on CD34+CD45dim cells

To address functional aspects associated to the expression of these new proteins, we focused our attention on MOSC-1, the expression of which had not been reported previously on cord blood derived CD34+CD45dim cells.

MOSC-1 (Moco Sulphurase domain containing protein-1) is a potentially secreted protein that contains a MOSC domain. This domain is predicted to be a sulfur-carrier domain that receives sulfur abstracted by the pyridoxal phosphate-dependent NifS-like enzymes, on its conserved cysteine, and delivers it for the formation of diverse sulfur-metal clusters [25].

**Figure 5:**
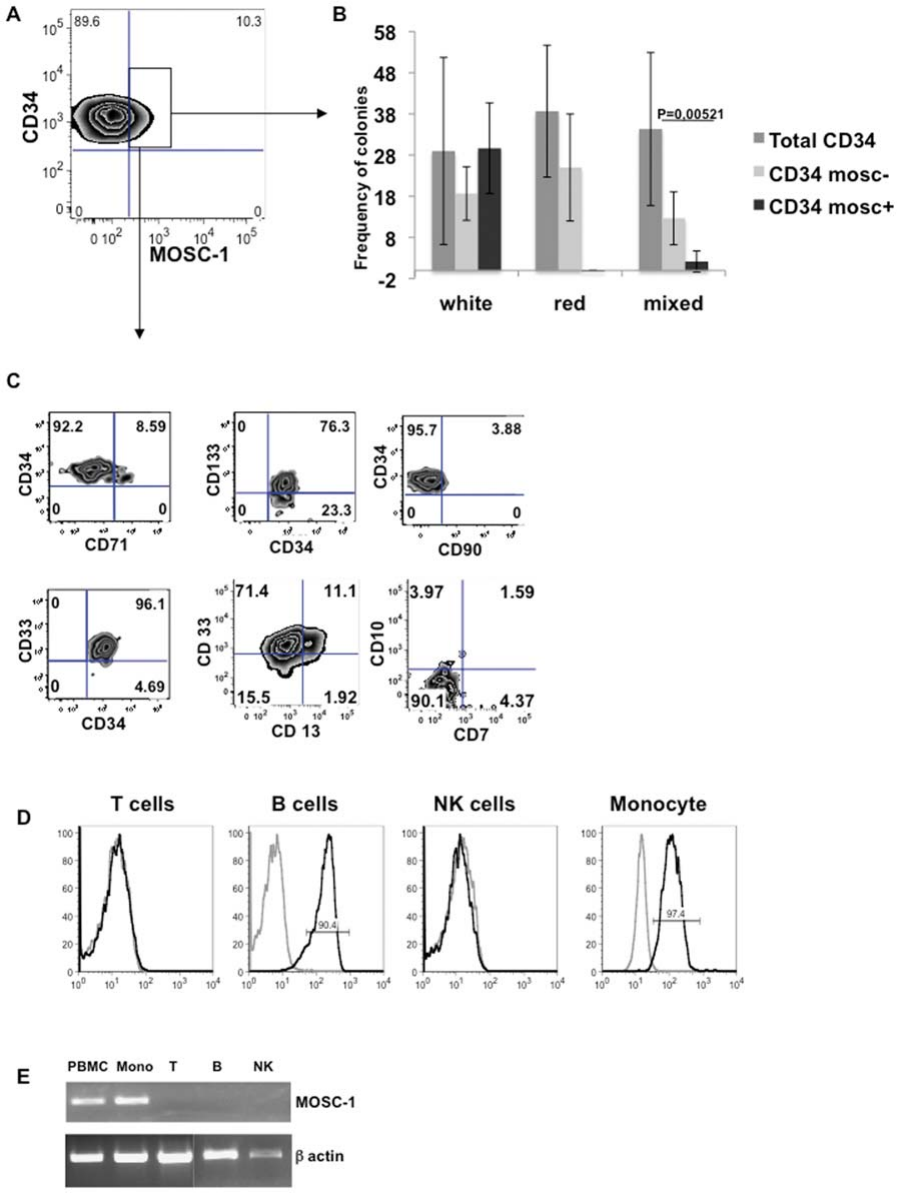
Pre-characterization of MOSC-1 expressing CD34+ cells. (A) Representative distribution of MOSC-1 on CD34+ cells. Cord blood mononuclear cells are stained with MOSC-1-specific serum diluted 1∶150. The analysis upon gating on CD34highCD45dim cells is shown. (B) CFC assay with MOSC-1 positive cells. CD34+MOSC-1+ and CD34+MOSC-1- cells were purified by Fluorescence Activated Cell Sorting. The purity of the populations used in the assays was >90%. The same number of cells from the two populations (100–500) were plated in methocult medium (Stem Cell Tech.) and incubated at 37°C for 14 days. Then the frequency of white, red and mixed colonies was calculated. The average of 5 independent experiments is shown. (C) Phenotype of CD34+ cells expressing MOSC-1. Cord Blood mononuclear cells were stained with anti MOSC-1 antiserum at 1∶150 dilution point after magnetic enrichment of CD34+ cells. All the samples were stained also with anti CD34 and anti CD45 mAbs and, in turn, with anti CD71, anti CD33, anti CD133, anti CD90, anti CD13, anti CD7 and anti CD10 mAbs. The expression of these markers is shown on the population of CD34+MOSC-1+ cells. An analysis representative of three independent experiments is shown. (D) MOSC-1 distribution on PBMC. PBMC from healthy donors were stained with anti MOSC-1 antiserum at 1∶150 dilution points. The samples were stained also with anti CD3 (T cells), anti CD19 (B cells), anti CD56 (NK cells) and anti CD14 (monocytes) mAbs to gate the correct subpopulation. An analysis representative of 5 different experiments is shown. In all the staining (C,D) serum from not immunized mice was used as negative control. (E) MOSC-1 RT-PCR on PBMC. RNA from PBMC and from the indicated magnetically purified subpopulation (Purity >99%) was retro-transcribed and amplified with MOSC-1 specific primers. Beta actin gene was amplified as positive control.

To assess whether the presence of MOSC-1 conferred peculiar functions to the CD34+CD45dim cells, we separated CD34+ cells expressing MOSC-1 (Figure 5A shows a representative plot) by FACS and performed a colony forming cell (CFC) assay to establish their differentiation capacity in comparison to the MOSC-1 negative counterpart. Therefore, the FACS sorted cells were plated in a semi-solid medium in the presence of a cocktail of growth factors (SCF, Flt3L, IL-6, GM-CSF, IL-3 and EPO) capable of sustaining proliferation and differentiation of different hematological lineages. After 14 days of culture, erythroyd progenitors would generate BFU (red) colonies, myelo-granulocytes progenitors would generate CFU-GM, CFU-G, CFU-M (white) colonies and the more immature cells would generate CFU-GEMM (mixed) colonies.

Figure 5B shows that CD34+ cells expressing MOSC-1 generated almost exclusively white colonies, while the frequency of mixed colonies is consistently reduced in comparison with MOSC-1 negative and total CD34+CD45dim cells. Finally the entire sample analyzed produced a negligible amount of red colonies with a significant frequency difference toward total CD34+CD45dim cells. These results suggest a commitment of MOSC-1 positive cells toward myeloid lineage. Moreover, a further phenotypic analysis performed on MOSC-1 positive CD34+ cells (Figure 5C) showed CD133 and CD33 co-expression together with no expression of CD7 and CD10, and a partial expression of the myeloid marker CD13, thus suggesting possible mono-granulocytic commitment. This possibility is consistent with the reactivity of MOSC-1 antiserum on peripheral blood monocytes (Figure 5D). Although Figure 5D shows a MOSC-1-specific reactivity also toward B-lymphocytes, RT-PCR analysis performed on purified PBMC subpopulations (Figure 5E) indicated that MOSC-1 transcript is present only in monocytes. Moreover, differentiation assays on OP9 cells demonstrated that CD34+ cells expressing MOSC-1 are not able to generate B cells (Figure S4). Since MOSC-1 is potentially secreted, it is possible that the protein produced and secreted by monocytes binds a receptor on B cells. Further studies are necessary to understand the reactivity of MOSC-1 positive serum on B cells, however the results that we have obtained with MOSC-1 expressing CD34+CD45dim cells are consistent with a monocyte commitment of this cell subset.

We conclude that using this antibody library we could not only identify new molecules expressed on subsets of cell populations of interest, but could also demonstrate a correlation between a phenotype and the functional commitment of the newly identified cell subset.

## Discussion

In this study we have described a library of mouse polyclonal antisera specific for linear epitopes of poorly known human proteins that were predicted to be either transmebrane or secreted. We have shown that with this library it is possible to analyze the cell surface of hematopoietic cells by flow cytometry and to identify new subsets of both mature and hematopoietic cells. This library is versatile—it can be used to screen any cell or tissue of interest—and allows screening of a large (1287) repertoire of “neglected” human proteins for those that mark specifically new subsets within apparently homogeneous cell populations.

The identification of new proteins on cells or tissues is generally based either on transcriptomics, *i.e.*, the assessment of mRNA expression profile, or on proteomics, *i.e*., the direct identification by mass spectrometry of proteins separated by 2D gels or liquid-based separation methods. Transcriptomics allows analyzing and compare large amount of samples at the same time [12], but poses the problem of the correlation between mRNA and protein expression levels. Proteomics is very informative but poses the problem of the complexity of the approach that makes it not suitable for high throughput screenings. In both cases, it is impossible to assess whether differences in the expression levels of genes or proteins occur in all of the cells analyzed or in a subset of them. It is therefore difficult to study those cell subsets or lineages that are poorly represented within a population. Our goal was to study hematopoietic cell subsets by flow cytometry and we therefore opted for an approach based on the direct identification of proteins with an antibody library.

We elicited our antibody library using proteins expressed in bacteria as immunogens. On one hand, antibodies raised against human proteins expressed in bacteria and purified from inclusion bodies are not ideal for the identification of human proteins present on the cell membrane, as these quite often undergo posttranslational modifications and structure conformations, which are generally lost when the protein is expressed in bacteria. Consequently, monoclonal antibodies specific for human proteins expressed in bacteria have the limiting factor of the number of antibodies that need to be screened to find the ones that recognize the human proteins in their native form. We therefore utilized polyclonal antisera that include a combination of specificities in the same sample. On the other hand, the expression of human antigens as his-tag proteins in bacteria has several practical advantages such as the higher throughput-working pipeline, the higher amount of proteins produced and the higher homogeneity of the different batches.

Since these types of antibodies quite often recognize on the native form of human proteins only primary sequence structure, *i.e.*, linear epitopes, and frequently they are even specific for epitopes that are not present on the “real life” proteins, it was important to obtain a proof of concept that our approach was suited to identify proteins expressed on human cell membranes. Therefore, we produced antisera specific for twenty well characterized proteins known to be present on hematopoietic cells and showed that we could identify 60% of known proteins on the surface of living PBLs by flow cytometry and 85% of known proteins in fixed and embedded lymphoid tissues by IHC. A likely explanation for the superiority of IHC versus flow cytometry with these antisera relies on the nature of the antigens used to immunize mice. Indeed, human proteins on fixed and embedded cells, rather than on living cells, are likely to share more “denatured” epitopes with the same human proteins expressed in bacteria and purified as inclusion bodies. Another possible explanation for the IHC superiority is that fixed cells exposes also the cytoplasm making more antigens available for the antisera, while by flow cytometry only surface proteins are detected. However, there were three main reasons that made the flow cytometry more suited for our purposes: i) the easier access to blood samples rather than lymph node biopsies; ii) the possibility to perform multiple color staining on the cells of interest; iii) the possibility to gate and analyze fractions of very rare cells subsets (<1–2%) which would pass mostly undetected in a screening performed by IHC.

Remarkably, a large majority of the newly identified proteins are expressed on a fraction of B cells. This is somehow expected since a lower number of B cell markers have been characterized as compared to T cell markers. For a long period of time CD4+ T cells have been considered the “master” regulators of the immune responses [26] and a lot of functionally distinct T helper or regulatory subsets have been described and characterized [27], [28], [29], [30], [31], [32], [33], [34]. B cells were generally considered antibody- producing effector cells and a combination of few surface markers was used to discriminate between human naïve B cells, memory B cells (central memory) and antibody-producing plasma cells (effector memory) [35]. However, B cells are more heterogeneous than previously thought. Although usually overshadowed by the production of antibodies, the ability of B cells to play important antibody-independent functions (antigen presentation, T cell and Dendritic cell regulation and cytokine and chemokyne production) is well documented [36]. Through these functions B cells can profoundly influence the formation and organization of secondary lymphoid tissues and T cell development, activation and function [36]. Moreover, antibody-independent B cell functions can contribute either to the development or to the prevention of autoimmune diseases [37]. It seems reasonable to assume that a larger amount of functionally distinct subsets contribute in various ways to antibody-independent functions of B cells and that such subsets could be defined by the expression of few proteins poorly characterized so far, that we have identified with our polyclonal antisera library.

In the field of stem cells, the paucity of surface markers, allowing the separation of stem cells subpopulations with a specific fate, represents the major problem in stem cell based therapies. Thus, the identification of such new stem cell markers would significantly improve their use in therapy. In the present study we aimed at identifying new hematopoietic stem cells subsets. Hematopoietic stem cell (HSC) transplantation is, nowadays, the only widely used stem cell-based therapy [38], [39], [40]. Even though HSCs have been extensively studied in the last 30 years and a number of lineage-specific markers have been identified, the need of new subsets identification to better understand the hematopoiesis mechanisms is still strong. With our screening we have identified the protein MOSC-1 on a subset of cord blood derived CD34+CD45dim cells as well as on monocytes, where its expression was revealed also by gene expression profile. We have performed a phenotypic characterization of CD34+ cells expressing MOSC-1 and we have purified by FACS sorting the fraction of MOSC-1 positive cells that generated almost exclusively CFU-G and CFU-M colonies in CFC assays. Our data are not sufficient to demonstrate that CD34+ cells expressing MOSC-1 are fully committed monocytes progenitors. In vivo studies with both MOSC-1 positive and negative CD34+ cells would be necessary to conclude that MOSC-1 expressing cells generate monocytes, but the extremely low number of highly purified cells that we can get by FACS sorting makes these experiments almost impossible. De Bruin and colleagues describes the possibility to induce in vitro monocytes differentiation from myeloid progenitors in the presence of interferon-gamma [41]. Also these kind of experiments could help to demonstrate whether MOSC-1+ CD34+ cells are able to generate monocytes, however the complete characterization of hematopoietic progenitors is not the point we wanted to made in this paper. In fact, the goal of this work is to demonstrate the usefulness of our antisera library to identify new cell subsets and, in our opinion, our results are sufficient to suggest that MOSC-1 is a marker defining the monocytes progenitors therefore identifying a new functional subset of CD34+CD45dim cells.

In conclusion, we generated a library of polyclonal antisera specific for poorly characterized human surface proteins. This library is a powerful discovery tool. In fact, our sera allow not only to identify new molecules expressed on different cell subsets, but also to perform a phenotypic and functional pre-characterization of the newly identified cell subset. Moreover, we were able to discover the expression on cord blood CD34+ cells of new proteins, not previously outlined by other methods, indicating that we have developed a very sensitive approach particularly appropriate when working with poorly represented cells.

## Supporting Information

Data S1 **Paper describing the generation and validation of the antisera library used in this study.**
(PDF)

Figure S1 **IHC analysis with sera specific for well-known proteins.** Sections of human lymph nodes were pretreated with an antigen retrieval solution and were then incubated with the indicated antisera. Detection steps were done using a commercially available kit according to the manufacturer instructions. Peroxidase activity was developed with 3-3-diaminobenzidine-copper sulfate to obtain a brown-black end product. A) anti CD2, B) anti CD3 gamma, C) anti CD8 alpha, D) anti CD8 beta, E) anti CD72, F) anti CD69.
(TIFF)

Figure S2 **Results of sera screening by FACS on PBLs and cord blood cells.** A) FACS analysis of sera positive on PBLs. PBLs were stained with the indicated sera. The samples were also stained with anti CD3, anti CD19 and anti CD56 mAbs to analyze the sera reactivity upon gating on the different subpopulations. B) PBMCs are treated for 24 hours with 1 µg/ml of PHA. After the treatment both un-stimulated and stimulated cells are stained with KRTCAP-3-specific serum. C) FACS analysis of sera positive on cord blood cells. Cord blood mononuclear cells are stained with the indicated sera. The samples are stained also with anti CD45 and anti CD34 mAbs to perform the analysis upon gating on CD34high CD45dim cells. In all the cases the average with the relative standard deviation of five different donors is shown for each serum.
(TIFF)

Figure S3 **Assessment of antisera specificity on Hela transfected cells by FACS analysis.** Hela cells were transfected with a myc-tag version of the proteins identified with the sera library. At 24 hours from the transfection cells were stained with the indicated sera as described in Methods section. As negative control un-transfected wt Hela cells stained with the same antisera were used. The FACS analysis for the CRISP-1 and MOSC-1 proteins is not shown since the two proteins are secreted and mouse antisera cannot be used in intra cellular staining because of the high background.
(TIFF)

Figure S4 **Differentiation assay on OP9 cells.** Because of the reactivity of MOSC-1-specific antiserum on B cells, we have tested the capability of CD34+ cells expressing MOSC-1 to generate mature B cells upon differentiation on OP9 stromal cells. MOSC-1 positive and negative cells were sorted by FACS (purity >98%) and analyzed for the up-regulation of the B cell marker CD19 upon 14 days of culture on OP9 cells. 500 cells were plated for each condition. MOSC-1 positive cells were unable to generate B cells in this assay. This result is in agreement with the hypothesis that MOSC-1 is expressed only by mature monocytes and binds a receptor on B cells.
(TIFF)
