# Supplementary material for: Identification of New Hematopoietic Cell Subsets with a Polyclonal Antibody Library Specific for Neglected Proteins
Source: PLoS One. 2012 Apr 4;7(4):e34395. doi: 10.1371/journal.pone.0034395 (PMC3319577; doi:10.1371/journal.pone.0034395)
Supplement: Data S1 — Paper describing the generation and validation of the antisera library used in this study. (PDF) [file pone.0034395.s001.pdf]

A novel polyclonal antibody library for expression profiling of poorly characterized, membrane and secreted human proteins

Renata Grifantini[1]*, Massimiliano Pagani[2#], Andrea Pierleoni[1], Alberto Grandi[1], Matteo Parri[1], Susanna Campagnoli[1], Piero Pileri[1], Davide Cattaneo[2], Elena Canidio[2], Angela Pontillo[2], Elisa De Camilli[3], Renzo Nogarotto[1], Sergio Abrignani[1a], Giuseppe Viale[3b], Paolo Sarmientos[1,2], Guido Grandi[1c]

[1] Externautics S.p.A. Via Fiorentina 1, 53100 Siena, Italy

[2] PRIMM; Via Olgettina 58, 20132 Milan, Italy

[3] European Institute of Oncology, Via Ripamonti 483, 20141 Milan, Italy

[1a] Advisory Board, Externautics SPA and INGM, Via Francesco Sforza 28, 20122, Milan Italy

[3b] Advisory Board, Externautics SPA and European Institute of Oncology

[1c] Advisory Board, Externautics SPA and Novartis Vaccines & Diagnostics

[#] Present address: INGM, Via Francesco Sforza 28, 20122 Milan Italy

Renata Grifantini and Massimiliano Pagani contributed equally to this work

*Corresponding author:

Renata Grifantini, PhD

Via Fiorentina, 1 53100 Siena, Italy

Phone:+39 0577 1916287

Fax: +39 0577 43444

e-mail: renata.grifantini@externautics.com

Running Title: YOMICS™ antibody library against membrane/secreted human proteins

SUMMARY

Here we present the YOMICS™ antibody library, a new collection of 1559 murine polyclonal antibodies specific for 1287 distinct human proteins. This antibody library is specifically designed to target marginally characterized membrane-associated and secreted proteins. The YOMICS™ library was generated against human proteins annotated as transmembrane or secreted in GenBank, EnsEMBL, Vega and Uniprot human genome databases, and described in no or very few dedicated PubMed-linked publications (0-10 dedicated papers). *E. coli* recombinant forms of the selected proteins were expressed, purified, and used to raise antibodies in the mouse. The capability of YOMICS™ antibodies to specifically recognize their target proteins either as recombinant form or as expressed in cells and tissues was confirmed in different experimental contexts, including Western blot, FACS, confocal microscopy and immuno-histochemistry (IHC). Moreover, to show the applicability of the library in immuno-histochemistry (IHC), five YOMICS™ antibodies against proteins either known to be expressed in some cancers or homologous to tumor-associated proteins were tested on tissue microarrays carrying tumor and normal tissues from breast, colon, lung, ovary and prostate. A consistent differential expression in cancer was observed. Our results indicate that the YOMICS™ antibody library is a useful tool for systematic protein expression profile analysis that nicely complements the already available commercial antibody collections.

INTRODUCTION

Membrane-associated and secreted proteins play a crucial role in many cellular and physiological processes, being mediators of material and information transfer between cells and their environment and within cellular compartments. Therefore, it is not surprising that their abnormal expression, both in quantitative and qualitative terms, including alterations in post-translational modification and gene mutations, is frequently associated with many disorders such as malignancies, cardiopathies, endocrine and neurodegenerative diseases (1). For these reasons, the study of membrane and secreted proteins in healthy and disease state has become an extremely active field of medical research aimed at identifying new diagnostic and therapeutic targets.

In spite of the intense research efforts of the last several years, a detailed characterization of membrane-associated and secreted proteins, which represent 30% of all human proteins, are far from being completed; a large fraction of them have their cellular location not experimentally confirmed yet, and approximately 30-40% still have an unknown function, according to gene annotation currently available in public databases (SwissProt). This lack of knowledge is mostly due to the enormous challenge the definition of secretomes and membranomes of the very many human tissues poses from a technical standpoint.

One of the possible approaches for proteomes and sub-proteomes characterization is the use of immunochemical techniques. Theoretically, if antibodies specific for all human proteins were available, one could use them in a variety of powerful experimental settings, including FACS analysis and confocal microscopy of cultured and ex vivo-isolated cells, Western Blot of cell culture supernatants and body fluids, immunohistochemistry of human tissues. For instance, in 2003 the Human Protein Atlas (HPA) project was launched which aims at systematically exploring the proteomes in 46 healthy human tissues, 20 tumors and 47 human cell lines (2-5). In essence, the HPA approach is based on (i) the high throughput expression in E. coli of all annotated human proteins, (ii) the production of rabbit polyclonal antibodies against each recombinant protein, (iii) the expression analysis of each protein by screening the antibody library on tissues and cell lines using immunohistochemistry and confocal microscopy. As for December 2010, the project has released expression data on 10,118 proteins (http://www.proteinatlas.org/) and the prediction is to complete a first draft of the Human Proteome Atlas in 2014 (2). In addition to ATLAS antibodies, polyclonal and monoclonal antibodies against a long list of human proteins are commercially available from several companies, including Abgent, Santa Cruz

Biotechnology, Inc., Aviva Systems Biology, Novus Biologicals, Abnova Corporation, LifeSpan BioSciences, Abcam, and Sigma-Aldrich. It is interesting to note that none of the available libraries was constructed to specifically target membrane-associated and secreted proteins with the result that for a large fraction of these important protein families specific antibodies are not available yet. Therefore we decided to embark in a project very similar methodologically to the HPA project but exclusively focused on the analysis of membrane and secreted proteins. More specifically, among these classes of proteins we prioritized those that, on the basis of the available annotation and the published scientific literature, we classified as hypothetical and/or marginally characterized. Furthermore, differently from other libraries that have used rabbit immunization for polyclonal antibody production, our polyclonal antibody library was generated in mice.

In this work we describe the selection of genes encoding membrane and secreted proteins and the subsequent production of protein and antibody libraries consisting of 1559 elements (thereinafter referred to as *YOMICS*^{TM} protein and antibody libraries). We also provide data that validate the specificity of the antibody library as judged by the capacity of a subset of antibodies to specifically recognize the corresponding proteins (i) purified from E. coli, (ii) expressed in transfected cells, and (iii) expressed in human tissues. Finally, we provide some examples of how the antibody library can be exploited for the identification of tumor markers using immunohistochemistry analysis.

Experimental procedures

Gene selection and subcellular localization and functional annotation of selected proteins

A local snapshot of the currently available human proteome was built by merging all the protein sequences available from four public resources: GenBank, Ensembl, Vega and Uniprot. Proteins sharing exactly the same aminoacidic sequences were treated as a single record. The merged human proteome comprises more than 71000 unique amino acidic sequences corresponding to more than 21000 protein coding genes. Since public annotation of signal peptides (SignalP) and transmembrane helix (TMHMM) are available for almost all the protein sequences, 7902 genes were selected, each one encoding for proteins with a secretion signal peptide or at least one transmembrane helix. These genes were further ranked on the basis of the number of scientific papers associated with them. For each gene associated scientific papers were retrieved from the NCBI 'gene2pubmed' file (available at ftp://ftp.ncbi.nlm.nih.gov/gene/DATA/gene2pubmed.gz) as described in the Results.

To assess the putative subcellular localization, all the proteins encoded by the library genes were predicted with a suite of state-of-the-art localization predictors. In the first prediction step, ENSEMBLE (6) was used to discriminate between globular and transmembrane proteins. The most probable subcellular localization of target proteins was then predicted with MemLoci (7) and BaCelLo (8), for transmembrane and globular proteins respectively.

For functional annotation, the target proteins were aligned with BLAST (9) against the manually curated SwissProt database (release 57). For each target protein, if a hit was found reporting a sequence identity higher than 60% and an Evalue lower than 10^{-5}, all the GeneOntology (GO) terms associated to the corresponding SwissProt entry were retained. In order to build pie charts indicative of target proteins functions, GO terms belonging to "Molecular Functions" and "Biological Process" ontologies were remapped to have a node rank of level 3 in the corresponding ontology tree. For each target protein only one annotation was used to compute pie charts.

Survey of available commercial Antibodies for YOMICS selected genes

The online commercial portal www.biocompare.com is currently indexing all the major antibodies library including those coming from Protein Atlas/Signam-Aldrich, Abnova, LifeSpan BioSciences, Abcam, Novus Biologicals, Santa Cruz Biologicals Abgent, Aviva Systems Biology, LifeSpan BioSciences and many more. For each gene of the YOMICS library a list of available commercial antibodies was built by querying the Biocompare database. Each gene symbol was used as antigen name in the Biocompare

antibody search, and available antibodies in the result page were locally stored for further analysis. Survey results are updated to January 2011.

Generation of recombinant human protein antigens and polyclonal mouse antibodies

The entire coding region or suitable fragments of the genes encoding the target proteins, were designed for cloning / expression using bioinformatics tools. Where present, the leader sequence for secretion was replaced with the ATG codon to drive the expression of the recombinant proteins in the cytoplasm of E. coli. For cloning, cDNAs were generated by reverse transcription from total RNAs (Euroclone) of human tissues (bone marrow, fetal brain, placenta and testis) and pooled. Gene fragments were amplified from cDNA pools using specific primers (designed using Primer3 Software, Premier Biosoft International) so as to fuse a 10 histidine tag sequence at the 3' end, annealed to in house developed vectors, derivatives of vector pSP73 (Promega) adapted for the T4 ligation independent cloning method (10) and used to transform *E.coli* NovaBlue cells recipient strain. *E. coli* transformants were plated onto selective LB plates containing 100 µg/ml ampicillin (LB Amp) and positive E.coli clones were identified by restriction enzyme analysis of purified plasmid followed by DNA sequence analysis. For expression, plasmids were used to transform BL21-(DE3) *E.coli* cells and BL21-(DE3) E. coli cells harbouring the plasmid were inoculated in ZYP-5052 growth medium (11) and grown at 37°C for 24 hours. Afterwards, bacteria were collected by centrifugation, lysed into B-Per Reagent containing 1 mM MgCl2, 100 units DNAse I (Sigma), and 1 mg/ml lysozime (Sigma). After 30 min at room temperature under gentle shaking, the lysate was clarified by centrifugation at 30.000 g for 40 min at 4°C.

All proteins were purified from the inclusion bodies. Briefly *E.coli* BL21-(DE3) cells harboring the plasmid were inoculated in 50 ml of ZYP-5052 growth medium (11) and grown at 37°C for 24 hours. Afterwards, bacteria were harvested by centrifugation and lysed into 10 ml of B-Per Reagent (Pierce) containing 1 mM MgCl2, 10 units/ml of DNAse I (Sigma), and 1 mg/ml Lysozime (Sigma). After 30 min at room temperature under gentle shaking the insoluble inclusion bodies were collected by centrifugation at 30.000 x g for 40 min at 4°C.

To solubilize inclusion bodies, the pellet was resuspended in 10 ml of 6M guanidine hydrochloride, 1 mM Tris(2-carboxyethyl)-phosphine hydrochloride (TCEP) (Pierce), 40 mM Tris-HCl pH 8. After clarification by centrifugation at 30.000 g for 30 min, the supernatant was loaded on 0.5 ml columns of Ni-activated Chelating Sepharose Fast Flow resin (GE-Healthcare). After wash of the resin

with 10 column volumes of 6M urea, 60 mM imidazole, 0.5M NaCl, 1 mM TCEP, 50 mM TRIS-HCl pH 8, recombinant proteins were eluted with the same buffer containing 500 mM imidazole. Proteins were analysed by SDS-Page and their concentration was determined by Bradford assay using the BIORAD reagent (BIORAD) with a bovine serum albumin standard according to the manufacturer's recommendations.To generate antisera, the purified proteins were used to immunize CD1 mice (6 week-old females, Charles River laboratories, 5 mice per group) subcutaneously with 3 protein doses of 20 micrograms each, at 2 week-interval. Freund's complete adjuvant was used for the first immunization, while Freund's incomplete adjuvant was used for the two booster doses. Two weeks after the last immunization animals were bled and sera collected from each animal was pooled.

In-gel Protein Digestion and MALDI-TOF Mass Spectrometry Analysis

The identity of recombinant affinity purified proteins was further confirmed by tandem mass spectrometry (MS/MS).Protein spots were excised from the gels, treated with with 50 mM ammonium bicarbonate (Fluka Chemie AG, Buchs, Switzerland) in 50% acetonitrile (J. T. Baker Inc.), de-hydrated once with pure acetonitrile and air-dried. Dried spots were digested for 2 h at 37 °C in 12 μl of 0.012 μg/μl sequencing grade modified trypsin (Promega, Madison, WI) in 5 mM ammonium bicarbonate. After digestion, a 0.6 μl was loaded on a matrix PAC target (Prespotted AnchorChip 96, set for proteomics, Bruker Daltonics, Bremen, Germany) and air-dried. Spots were washed with 0.6 μl of a solution of 70% ethanol, 0.1% trifluoroacetic acid. Mass (MS) spectra were acquired on an Ultraflex III TOF/TOF mass spectrometer (Bruker Daltonics) in reflectron, positive mode in the mass range of 900–3500 Da. Also MS/MS spectra were acquired. Spectra were externally calibrated by using a combination of standards pre-spotted on the target (Bruker Daltonics). MS and MS/MS spectra were analyzed with FlexAnalysis (FlexAnalysis version 3.0, Bruker Daltonics). Monoisotopic peaks were annotated with FlexAnalysis default parameters and manually revised. Protein identification was carried from the generated peak list using the Mascot program run on a public database (NCBI nr Homo sapiens, release 20100616 or SwissProt Homo sapiens release 2010_07.

Tissue Microarray profiling by immunohistochemistry

The analysis of the antibodies capability to recognize their target proteins in tumor samples was carried out by Tissue Micro Arrays (TMA). A tissue microarray was prepared containing 100 formalin-fixed

paraffin-embedded cores of human tissues from patients affected by colorectal cancer, ovarian cancer, breast cancer, lung cancer, prostate cancer and corresponding normal tissues and analyzed using the specific antibody. Briefly, each TMA included 50 tumor tissue cores representative of 5 different well pedigreed patients for each of the 5 cancer types, and an equal number of normal tissue cores from the same patients. In total, the TMA included 10 tumor cores per each tumor class and 10 normal tissue cores per each organ (equal to two tumor samples and 2 normal tissues from each patient) to identify promising target molecules differentially expressed in cancer and normal tissues. The direct comparison between tumor and normal tissues of each patient allowed the identification of antibodies that differentially stain tumor cells and provide indication of target expression in the tumor under investigation.

All formalin-fixed, paraffin-embedded tissues used as donor blocks for TMA production were selected from the archives at the IEO (Istituto Europeo di Oncologia, Milan, Italy). Corresponding whole tissue sections were examined to confirm diagnosis and tumour classification, and to select representative areas in donor blocks to be cored. Normal tissues were defined as microscopically normal (non- neoplastic) and were generally selected from specimens collected from the vicinity of surgically removed tumors. The TMA production was performed essentially as previously described (12). Briefly, a hole was made in the recipient TMA block. A cylindrical core tissue sample (1 mm in diameter) from the donor block was acquired and deposited in the recipient TMA block. This was repeated in an automated tissue arrayer “Galileo TMA CK 3500" (BioRep - Milan) until completion of the TMA design. TMA recipient blocks were baked at 42 <0>C for 2 h prior to sectioning. Two to 3 micron thick sections were cut from the TMAs and placed onto poli-L-lysinated glass slides for immunohistochemical analysis. Automated immunohistochemistry was performed as previously described (13). Briefly, the glass slides were heated for 30’ min at 60°C, de-paraffinized in xylene (2 x 15 min) using the Bio-Clear solution (Midway. Scientific, Melbourne, Australia), and re-hydrated in graded alcohols. For antigen retrieval, slides were immersed in 0.01 M Na-citrate buffer, pH 6.0 at 99°C for 30 min, placed in an automatic immunostainer (Autostainer (R) Dako, Glostrup,Denmark)) and endogenous peroxidase was initially blocked with 3% H2O2, for 5 min. Slides were then blocked in Dako Wash Buffer containing 5% Bovine serum albumin (BSA) and subsequently incubated with the different mouse antibodies for 30’ (dilution 1:200 in Dako Real ^{TM} dilution buffer). After washing with Dako wash buffer, slides were incubated with the goat anti-mouse peroxidase conjugated Envision(R) detection reagent (Dako) for 30 min at room temperature. Finally, diaminobenzidine and the substrate chromogen (Dako) was used to visualize the

reaction and Harris hematoxylin (Sigma Aldrich) was used for counterstaining. The slides were mounted with Pertex(R) (Histolab).The staining results have been evaluated by a trained pathologist at the light microscope, and a IHC score was assigned corresponding to the the percentage of immunostained cells (from 0 to 100%) multiplied by the intensity of staining (from 1 to 3). The individual values and the combined score (from 0 to 300) were recorded in a custom-tailored database, that also included the subcellular localization of the immunoreactivity (membranous, cytoplasmic, nuclear). Digital images of the immunocytochemical findings have been taken at a Leica DM LB light microscope, equipped with a Leica DFC289 color camera.

Protein expression analysis in tumor cell lines by Western blot and confocal microscopy

Human epithelial cells were obtained from the ATCC collection and cultured in under ATCC recommended conditions. For transfection experiments, protein encoding plasmids were generated by PCR amplifying the full length coding regions of selected genes with specific primers pairs and PCR products were cloned into plasmid pcDNA3 (Invitrogen) and sequence verified. HeLa cells (400,000 per well, in 6 well-plates) were transiently transfected with 4 micrograms of the resulting plasmids and with the empty vector as negative control (mock) using the Lipofectamine-2000 transfection reagent (Invitrogen). Twenty four hours after transfection, cell were collected for immunoblot analysis.

For immunoblot analysis sub-confluent cell mono-layers were detached with PBS-0.5 mM EDTA and lysed by several freeze-thaw passages in PBS-1% Triton. Total protein extracts were loaded on SDS-PAGE ($2x10^5$ cells/lane) and expression of target proteins was assessed by Western blot analysis using specific antibodies. Western blot was performed by separation of the protein extracts on pre-cast SDS-PAGE gradient gels (NuPage 4-12% Bis-Tris gel, Invitrogen) under reducing conditions, followed by electro-transfer to nitrocellulose membranes (Invitrogen) according to the manufacturer's recommendations. The membranes were blocked in blocking buffer composed of 1x PBS-0.1% Tween 20 (PBST) added with 10% dry milk, for 1 h at room temperature, incubated with the antibody diluted 1:2500 in blocking buffer containing 1% dry milk and washed in PBST-1%. The secondary HRP-conjugated antibody (goat anti-mouse immunoglobulin/HRP, Perkin Elmer) was diluted 1:5000 in blocking buffer and chemiluminescence detection was carried out using a Chemidoc-IT UVP CCD camera (UVP) and the Western lightning™ cheminulescence Reagent Plus (Perkin Elmer), according to the manufacturer's protocol.

For confocal microscopy, cells were plated on glass cover slips and after 48 h were washed with PBS and

fixed with 3% formaldheyde solution in PBS for 20 min at RT. Then, after extensive washing in PBS, the cells were permeabilized with 0.01% BriJ96® (Fluka) and subsequently incubated with the appropriate antibodies overnight at 4°C (1:200). Cells were then stained with Alexafluor 488-labeled goat anti-mouse antibodies (Molecular Probes). DAPI (Molecular Probes) was used to visualize nuclei. The cells were mounted with glycerol plastine and observed under a laser-scanning confocal microscope (LeicaSP5).

RESULTS

Construction of the YOMICS libraries of human proteins and polyclonal antibodies

Gene selection and in silico annotation

To generate the antibody library, we selected proteins fulfilling the criteria of being a) predicted as integral membrane or secreted, and b) poorly characterized, as defined on the basis of a search in the published scientific literature available at time of the project start (2006). Protein selection was made *in silico* by integrating gene and protein data from four major sources: GenBank, Ensembl, Vega and Uniprot.

SignalP (http://www.cbs.dtu.dk/services/SignalP) and TMHMM (http://www.cbs.dtu.dk/services/TMHMM/) algorithms were used to select genes encoding proteins with signal peptides and/or one or more transmembrane helices. The resulting 7902 genes, which include those encoding proteins destined to all cellular membranes, were further ranked on the basis of the number of PubMed-linked scientific papers associated to each gene. Papers mentioning a specific gene/protein in the context of proteomic and microarray studies where more than 50 other genes/proteins are described were regarded as not gene-specific and therefore were not taken into account. This process led to the selection of 5086 genes which had been cited/described in less than 10 scientific publications. At present, we focused our attention on 1287 of these genes which, for their putative predicted function and cellular location (see below), were considered of particular interest. At the time of their selection, approximately 42% (547 genes) of the 1287 genes had no associated papers, 47% (604 genes) had 1 to 5 associated papers and the remaining 11 % (146 genes) had 5 to10 papers. We recently (September 2010) re-analyzed the 1287 gene pool to follow the progress in scientific knowledge over the period 2007-2010. Somehow surprisingly, we found that only 18 genes (1.5% of the entire library) are currently mentioned in more than 10 published papers.

The proteins encoded by the selected genes, including the different variants derived from alternative splicing, were analyzed *in silico* to obtain global statistics about their function and sub-cellular localization (6-8). As shown in Figure 1A, the localization analysis indicated that 61% of the proteins are predicted to

be transmembrane, 75% of which are assigned to the plasmatic cell membrane. Almost a quarter of the library proteins (23%) accounts for secreted proteins, while approximately 16% are soluble intracellular proteins. However, this last fraction includes protein variants derived from alternative mRNA splicing of genes which also have at least an additional transmembrane or secreted isoform.

GeneOntology (GO) terms (http://www.geneontology.org/) were used to obtain a detailed and updated functional annotation of the library. For each target protein GO terms were retrieved from the corresponding SwissProt entry (http://www.expasy.ch/sprot/) or, if feasible, very close homologues entries. Pie charts indicative of target proteins functions were built by reporting GO terms belonging to "Molecular Functions" (Figure 1C) and "Biological Process" (Figure 1B). Approximately half of the library is composed of proteins of unknown function, while the other half include proteins that on the basis of the GO analysis could be assigned to specific functional categories, among which receptor, transport, cell-to-cell related functions and immune responses are the most populated ones.

Generation of YOMICS protein and antibody libraries

Our cloning and expression strategy was based on the selection of proteins or fragments ranging from 100 to 500 amino acids in length and not carrying predicted transmembrane regions. The fragments were selected by analyzing the protein sequences encoded by the 1287 selected genes (including all annotated variants) and by identifying those the algorithms predicted to be outside the membrane with high scores and/or structural domains. For 214 genes more than one protein fragment were selected. In total, 1559 polypeptides were expressed in E. coli, 1075 of which corresponded to protein fragments and 484 corresponded to full length proteins (Table 1S). In addition, 20 genes encoding well characterized membrane associated proteins (Table I) were included in the cloning/expression/antibody production pipeline to be used as positive controls and to validate the overall approach.

DNAs were amplified by RT-PCR using, as templates, pools of commercially available RNAs derived from different human tissues and cloned into the p2N vector (see Materials and Methods), so as to generate recombinant protein fusions with a 10-histidine tag at the amino-terminus. E. coli clones were sequence-verified using gene specific primers able to anneal at the 5' and 3' terminus of each cloned gene fragment. Fragments carrying nucleotide mutations resulting in more than 3 amino acid substitutions were re-cloned. The 1559 recombinant proteins were affinity-purified by IMAC-chromatography from the inclusion bodies and their purity was estimated by SDS-PAGE and Coomassie Blue staining. As shown in Figure 2A representing the Comassie Blue staining of two gels in which the 20 control proteins (Table I)

and 20 proteins from the YOMICS library were resolved, the purification procedure led to protein preparations constituted by a major band (indicated by the arrow) with the expected electrophoretical mobility. Other protein bands were also often visible and in most of the case they represented aggregation/degradation products of the recombinant protein as confirmed by Mass Spectrometry analysis (see below). To further validate the robustness of the cloning/expression/purification process, 120 randomly selected recombinant proteins were resolved by SDS-PAGE and the prevalent bands visible in each lane after staining were excised from the gel and analyzed by Mass Spectrometry. All analyzed proteins corresponded to the expected ones and, as said above, most of the protein species of higher or lower masses derived from the cloned proteins (data not shown).

Groups of CD1 mice (five mice/group) were immunized with each protein and sera from each group were pooled together. In general, IgG titers ranging from 10,000 to 80,000 (serum dilution giving a OD_{450} value higher than 3-fold the pre-immune sera) were obtained, as assessed by ELISA using plates coated with the corresponding recombinant protein.

Validation of the YOMICS antibody library

a) Specificity of the YOMICS^{TM} antibody library

The YOMICS antibody library currently available consists of 1559 antibodies directed against 1287 distinct human proteins. Once completed, the library was subjected to an intense validation process. First, we verified the capability of YOMICS^{TM} antibodies to recognize the respective affinity-purified recombinant protein by Western blot. The antibodies corresponding to the 120 proteins confirmed by MS analysis (see above) were selected for this purpose. All tested antibodies were able to recognize protein bands of the expected molecular mass. Frequently, the antibodies also detected truncated or aggregated forms of the selected proteins, as verified by MS analysis of corresponding bands excised from the gels. Typical examples of SDS-PAGE and Western blot are shown in Figure 2 where control proteins (Table I) and 19 YOMICS^{TM} proteins were analyzed. Moreover, since all proteins used for immunization carried the His-tag peptide known to be immunogenic in mice, to make sure that protein recognition was mostly due to anti-protein antibodies and not to anti-His-tag antibodies, a panel of 10 sera was pre-adsorbed on a CNBr-activated Sepharose matrix coated with the His-tag peptide to remove the anti-His-tag-specific antibodies. Treated sera were then tested for specificity by Western Blot on membranes carrying the corresponding proteins as well as other 18 unrelated proteins. All tested antisera were able to detect their respective proteins while they showed negligible recognition of the unrelated proteins (Figure 2C).

The second validation analysis consisted in evaluating whether the antisera were able to recognize their respective proteins when expressed in mammalian cells after gene transfection. To this purpose, the coding sequences of ten genes were cloned in pcDNA3.1D mammalian expression vector and the derived protein-encoding plasmids were used to transiently transfect HeLa cells. Four of the ten protein sequences are predicted as secreted (FLST5, ANGPTL7, COLEC11 and VMO1), five are predicted as associated to intracellular membranes (HIGD2A, HIGD2B, C9orf46, C4orf32, FAM82A2) and the last one (E-Syt1) is predicted as localized at the plasma membrane. At 24h post-transfection, total cell lysates and cell culture supernatants were subjected to Western blot with the appropriate antisera. As shown in Figure 3A, protein species of expected size (from approximately 12 to 94 KDa) were specifically detected in transfected HeLa cells, while cells transfected with the empty vector showed marginal or undetectable signals. In line with the prediction, the four secreted proteins were mainly detected in the culture supernatants while the six membrane-proteins were exclusively detected in the cell extracts. Confocal microscopy analysis was also carried out to confirm protein localization of this latter group using a tridimensional (3-D) reconstruction of the XY confocal sections (Figure 3B). As expected, the anti- E-Syt1 antibody stained the plasma membrane of transfected cells, while antibodies against HIGD2A, HIGD2B, C9orf46, C4orf32, and FAM82A2 showed cytoplasmic granular/vesicle-like staining, suggesting that these proteins are associated to intracellular membranous structures. Empty plasmid-transfected cells showed negative staining.

b) Applicability of the YOMICS^{TM} antibody library in Immunohistochemistry (IHC)

We next assessed whether the antibody library is amenable for IHC analysis. To this aim, tissue microarrays carrying normal human lymph node tissues were analyzed using the 20 antibodies raised against the control proteins (Table I) and corresponding to surface markers of hematopoietic cells (according to NCBI annotation data at www. ncbi.nlm.nih.gov). Fifteen of the twenty tested antibodies (75%) did show membrane staining of the expected cells in human lymph nodes (Table 2). Examples are given in Figure 4 which reports pictures of lymph nodes sections stained with antibodies specific for recombinant CD2, CD3ε, CD69, CD40 and CD72. As shown in Figure 4, the antibodies specific for CD2 and CD3 (both markers of T lymphocytes) yielded a membranous staining of most T lymphocytes of the para-follicular region and of scattered T cells, while anti-CD69 antibodies (CD69 is expressed in activated T lymphocytes) generated a membraneous staining of a subpopulation of T lymphocytes within the germinal center and in the paracortical area. The antibody against CD40 (protein constitutively expressed

in antigen-presenting cells including dendritic cells, B cells and macrophages) did stain the lymphoid and accessory cells of the germinal center while the antibody specific for CD72 (mainly expressed by B cells) produced an intense immunostaining of the majority of B lymphocytes in the germinal center and follicular mantel and of scattered B cells.

In addition to antibodies specific for T and B cell markers, we also tested the binding ability of the antibody specific for Her2 (Table 1) following the same cloning/expression/purification strategy used for the generation of the YOMICS^{TM} protein and antibody libraries. Her2 is a well known surface-exposed tyrosine kinase over-expressed in 15-20% of breast cancer and associated with increased disease recurrence and worse prognosis. The specificity of the YOMICS^{TM} anti-Her2 mouse antibodies was tested on known Her2-positive and Her2-negative clinical specimens (as defined by both immunohistochemistry and FISH assays for gene amplification) from breast tumor cancer patients. IHC analysis resulted, as expected, in an intense circumferential membrane staining of the vast majority (>95%) of invasive cancer cells in all known-positive cases, whereas it did not stain any of the known-negative cases (Figure 4 and data not shown).

c) Use of the YOMICS library for expression profiling in tumor tissues

One potential application of the YOMICS^{TM} antibody library is the search of new tumor markers through the screening of surgical samples from cancer patients. To confirm the applicability of the library for this particular purpose, in addition to the Her2 antibodies described above, we also tested antibodies raised against 5 recombinant proteins, including two members of the Cystein-Rich Secretory Proteins family (CRISP3 and CRISPLD2), the Interferon-Induced Transmembrane Protein 3 (IFITM3) and two proteins of the carcinoembryonic antigen family (CEACAM4 and CEACAM8). These proteins were selected since known either to be over-expressed in specific tumors or to belong to the same family of well characterized tumor biomarkers. In particular, CRISP3 and IFITM3 are over-expressed in prostate and colon cancers, respectively (14-18). CRISPDL2 is homologous to CRISP3, while CEACAM4 and CEACAM8 are homologous to CEACAM5, whose expression increases in colon cancers and other malignancies (19, 20).The anti-CRISP3 antibody (Ab-1265-YOM) and the anti-IFITM3 antibody (Ab-633-YOM) of our YOMICS^{TM} library basically confirmed what already known. Ab-1265-YOM showed specific staining of prostate cancer and Ab-633-YOM showed moderate staining of colon cancer (Figure 5 and Table 2). Interestingly and somehow not expected, Ab-633-YOM also showed weak staining of lung and ovary tumors, while Ab-1265-YOM showed moderate staining of breast tumor, with no staining of any of the five

normal tissues (Figure 5 and Table 2). As far as the other three proteins are concerned, CRISPLD2 showed an interesting opposite behavior with respect to its homolog CRISP3: in fact the anti-CRISPLD2 antibody (Ab1065-YOM) recognized three of the five normal prostate tissues and none of the corresponding tumor specimens (Figure 5 and Table 2). The anti-CEACAM8 antibody intensively stained both cells and extracellular secretions of lung and colon cancers and moderately recognized breast cancer cells while no reactivity was observed in normal tissues with the exception of a weak recognition of normal colon tissue (Table 2). Finally, the anti-CEACAM4 antibody was immunoreactive on lung and colon cancers, with lack of staining in normal tissues. In colorectal cancer staining was confined to the extracellular secretions (Table 2).

In conclusion, the results here presented show that the YOMICS antibody library is amenable for high throughput IHC screening of tumor markers. Antibodies against recombinant Her2, IFITM3 and CRISP3, proteins known to have altered expression in specific tumors, showed tissue specificity profiles in line with published data. Furthermore, the library appears to be effective in the identification of new proteins with altered expression in human cancers, as indicated by the expression profiling of CRISPLD2, CEACAM4 and CEACAM8, proteins homologous to known cancer markers but whose involvement in tumor metabolism was not known.

DISCUSSION

Membrane-associated and secreted proteins are of major interest for clinical applications. Secreted proteins dominate the diagnostic field since being released in the body fluids can be exploited in the development of non invasive early diagnostic and prognostic assays. Typical examples are the cancer biomarkers Carcinoembrionic antigen (CEA), the Prostate specific antigen (PSA) and the Cancer Antigen 125 (CA125) generally used for diagnosis and monitoring of colorectal, prostate and ovary cancer, respectively. The global biomarker market is expected to reach 20.5 billion by 2014 (source Datamonitor). Similarly, membrane-associated proteins exposed on the outer surface of cells are ideal targets for therapeutic molecules, including monoclonal antibodies. In 2009 the global market of therapeutic monoclonal antibodies targeting surface proteins reached $ 40 billion. Based on the increased number of mAbs under investigation in several clinical studies (In 2015 2008, 193 monoclonal antibodies were under investigation in about 375 clinical trials, 63 % of which for oncology applications (source Datamonitor)) the estimated sales of monoclonal antibodies in 2015 are expected to reach $ 67.6 billion.

Despite the recent progress in this field, a large portion of human membrane/secreted proteins has not been adequately studied yet, mainly due to practical difficulties, such as low abundance and the absence of appropriate and easy-to-use investigation tools (21). As a matter of fact, at the time of submission of this work, 5086 out of the predicted 7902 membrane and secreted proteins (proteins having an N-terminal signal sequence and/or one or more transmembrane spanning regions) either have never been described in scientific publications or have been the object of a very limited number of specific studies (from one to less than ten dedicated papers). The YOMICS™ antibody library described in this study is a novel collection of mouse polyclonal antibodies specifically designed to follow the expression in human cells and tissues of poorly characterized transmembrane and secreted proteins.

The robustness of the protein and antibody YOMICS™ libraries was validated by a number of experimental analyses. First, each clone of the expression library was sequenced to confirm the identity of the amplified cDNAs and in case the insert did not correspond to the expected fragment or carried more than three amino acid substitutions, amplification and cloning were repeated. A negligible amount of genes required a second round of amplification and cloning, in line with the fact that (i) the Primer 3 software algorithm used for primers design proved to guarantee high selectivity, and (ii) the high fidelity polymerase very rarely introduced errors in the relatively short (< 1,000 bp) genes/gene fragments selected for amplification. Second, after purification, 120 randomly selected recombinant proteins were

run on SDS-polyacrylamide gels and proteins bands were subjected to Mass Spectrometry analysis. All proteins showed a band of the expected molecular weight, even though the degree of purity varied from more than 80% to approximately 30%, as determined by gel densitometric analysis (data not shown). However, most of the additional protein bands present in each protein sample represented degradation/aggregation products of the corresponding protein, as confirmed by Mass Spectrometry analysis. Therefore our random sampling of one tenth of the protein library (120 proteins out of 1287) confirmed the identity of each protein and indicated that the affinity purification process led to purification yields appropriate to generate an antibody library of sufficient specificity. To support this conclusion, after immunization, sera derived from the 120 test proteins were analyzed by Western Blot. All antibodies recognized the expected protein bands. Since His-tagged proteins are known to often induce antibodies against the histidine tail (approximately 70% of all polyclonal antibodies of the YOMICS^{TM} library contain antibodies specific for the His-TAG), the Western Blot analysis was also carried out with a group of randomly selected antibodies after pre-incubation with the His-Tag peptide to absorb the anti-His Tag antibodies. All sera recognized their respective proteins and no cross-reaction with other irrelevant proteins was observed.

Since the antibody library derives from unfolded and unglycosylated proteins expressed in E. coli, it is particularly suitable for those techniques, such as Western blot, confocal microscopy and IHC, where samples preparation leads to the exposition of linear epitopes. In particular, thanks to the development of the tissue microarray (TMA) technique, the library is a useful tool for the analysis of hundreds of different clinical samples in high throughput modalities. We have validated the application of the YOMICS library in IHC by analyzing the specificity of a group of antibodies against control proteins produced following the same platform technologies used for the construction of the YOMICS^{TM} library. The proteins included twenty well known markers of the hematopoietic tissue and three proteins over-expressed in specific tumors. Seventy eight percent of the sera (18 out of 23) correctly stained the expected cells/tissues when used at 1:100-1:200 dilutions. Interestingly, the pre-absorption step with the His-tag peptide was not necessary, indicating that anti-His-Tag antibodies did not cross-react with sufficient affinity with any human component, at least at the dilutions used.

As already pointed out, several antibodies libraries against a large fraction of human proteins are commercially available. In addition, the Human Protein Atlas (HPA) project is engaged in the preparation of a rabbit antibody library against all annotated human proteins and in the exploitation of the library to

define the protein expression profile of 46 human tissues and 20 different tumors by using IHC. As for December 2010, the expression of approximately 40% of all human proteins has been analyzed and new proteins are being characterized at a pace of 20 proteins/day. This impressive undertaking, together with the availability of a growing list of monoclonal and polyclonal antibodies might apparently weaken the value of the library here described. However, we believe that in fact our library is complementary rather than redundant to the other existing antibody libraries for a number of relevant reasons.

First, differently from the existing antibody libraries, the YOMICS library is specifically dedicated to the poorly characterized cell surface proteins and secreted proteins. As a result, our recent analysis indicates that 40% of the antibodies included in YOMICS library are not available from any of the commercial library, and 66% of the YOMICS proteins have not been analyzed yet by HPA (HPA release June 2010). Second, the strategies to generate antibodies against human proteins can vary substantially from library to library. In some cases peptides are used, in others different recombinant fragments have been selected (e.g. in approximately 75% of the proteins selected by both HPA and YOMICS™ different protein regions were selected to raise antibodies) and in others different expression systems (E. coli, Baculovirus, mammalian cells, etc.) have been utilized. Non overlapping results can be obtained using antibodies derived from different libraries. For instance, different fragments derived from the same protein might vary in immunogenicity, might elicit antibodies recognizing different splicing isoforms or might be unable to bind the corresponding proteins in biological samples due the presence of post-translational modifications. Third, while the YOMICS polyclonal antibody library has been generated in mice, most of the other libraries include either mouse monoclonal antibodies or rabbit antibodies. This substantial difference can lead to the generation of antibodies recognizing different epitopes within the same protein, with the result that the expression analysis may vary depending upon which antibody preparation is used. This is particularly true in high throughput IHC analysis of TMAs where, in general, to reduce the number of samples to analyze fixed antibody concentrations are normally used. IHC has in fact a small dynamic range. For instance, the commercially available DAKO monoclonal antibody against Her2 (the target of the most successful anti-cancer monoclonal antibody therapy so far) discriminates breast tumor from healthy tissues when used at dilutions between 1:200 to 1:1,600. At higher dilutions no staining is observed in either tissues, while at 1:200 dilution or below both tissues are stained and no difference in Her2 expression can be revealed. Considering that healthy breast cells express an average of 20,000 Her2 molecules on their surface while cancers cells can have as many as 2,000,000 Her2 molecules, one

can easily appreciate the narrow window within which antibodies can establish whether or not a specific protein is expressed in a given tissue and eventually discriminate its differential expression between healthy and disease state.

The importance of having different antibody libraries for an accurate determination of protein expression profiling in cells and tissues will be experimentally supported when expression data on several proteins using different antibody preparations will become available. At this stage, very preliminary indications are provided by the comparison of the expression profiles of the three proteins CRISPLD2, IFITM3 and CEACAM4, which have been investigated on colon, breast, lung, ovary and prostate healthy and cancer tissues with both YOMICS (this work) and HPA (HPA web site: www.proteinatlas.org) antibodies. Data comparison highlights some substantial differences (Table 2S, Supplementary). As far as CRISPDL2 is concerned, a protein known to be spontaneously released by human peripheral blood granulocytes and mononuclear cells in response to stimulation with LPS (22), the HPA anti-CRISPDL2 antibodies showed a moderate to strong staining both in malignant and normal cells of all five tissues. By contrast, the YOMICS antibodies, which were raised using a different domain of CRISPDL2, visualized the protein only in normal prostate tissue, while prostate tumor cells and all the other tested tissues were negative. Similar remarkable differences were observed when CEACAM4 expression was analyzed. The HPA polyclonal antibodies stained with comparable intensities both normal and tumor cells from breast, colon, lung and prostate while ovary tissue was negative. Conversely, the YOMICS™ anti-CEACAM4 antibodies showed a specific staining of lung and colon cancers, with no staining of the corresponding healthy tissues and no staining of normal and tumor tissues from ovary, prostate and breast. Finally, as far as IFITM3 is concerned, a protein reported as one of the crucial candidate genes for inflammatory bowel disease and whose over-expression was found in ulcerative colitis-associated cancers, oral squamous cell carcinoma, gastric and colorectal cancer cells (14-16, 23, 24), the HPA antibodies showed a positive staining in all five tumors as well as in normal lung, ovary and breast. By contrast, the YOMICS anti-IFITM3 antibodies specifically stained colon, breast and ovary tumors while no expression in prostate and lung tumors and in all five normal tissues was observed. Interestingly, both HPA and YOMICS™ anti-IFITM3 antibodies were able to detect IFITM3 in tumor cell lines by using confocal microscopy, but they showed different subcellular localizations. The HPA antibodies generated an intracellular staining whereas the YOMICS™ antibodies gave a surface staining, in line with the predicted localization of the protein (Figure 1S shows the IFITM3 surface exposure on the Ovcar 8 tumor cell line). Based on the above, the overall impression

is that HPA anti-IFITM3, -CEACAM4 and -CRISPDL2 antibodies, which were purified by affinity chromatography, are used at concentrations closed to the saturating ones for IHC. Such concentrations are surely appropriate to detect low levels of protein expression but might not be ideal to discriminate differences in expression levels. By contrast, the YOMICS antibodies, which are not purified, might have relatively low concentrations and, when used at 1:100 dilution, could detect proteins only if expressed at sufficiently high levels.

In conclusion, the data here described indicate that the YOMICS antibody library is a useful tool to study protein expression in human cells and tissues using different immunoassays, including IHC. The data also suggest that in studying protein expression pattern and tissue-specificity the use of different concentrations of more than one antibody preparation recognizing different epitopes/regions of the same target protein should be considered to avoid the generation of incomplete or partially misleading results.

Our future plan is to expand the YOMICS library with particular interest for antibodies targeting marginally characterized cell membrane and secreted proteins for which antibodies are not yet available in commercial collections.

Reference List

1. Sanders, C. R., and Myers, J. K. (2004) Disease-related misassembly of membrane proteins. Annu. Rev. Biophys. Biomol. Struct. 33, 25-51

2. Service, R. F. (2008) Proteomics. Proteomics ponders prime time. Science 321, 1758-1761

3. Uhlen, M., Bjorling, E., Agaton, C., Szigyarto, C. A., Amini, B., Andersen, E., Andersson, A. C., Angelidou, P., Asplund, A., Asplund, C., Berglund, L., Bergstrom, K., Brumer, H., Cerjan, D., Ekstrom, M., Elobeid, A., Eriksson, C., Fagerberg, L., Falk, R., Fall, J., Forsberg, M., Bjorklund, M. G., Gumbel, K., Halimi, A., Hallin, I., Hamsten, C., Hansson, M., Hedhammar, M., Hercules, G., Kampf, C., Larsson, K., Lindskog, M., Lodewyckx, W., Lund, J., Lundeberg, J., Magnusson, K., Malm, E., Nilsson, P., Odling, J., Oksvold, P., Olsson, I., Oster, E., Ottosson, J., Paavilainen, L., Persson, A., Rimini, R., Rockberg, J., Runeson, M., Sivertsson, A., Skollermo, A., Steen, J., Stenvall, M., Sterky, F., Stromberg, S., Sundberg, M., Tegel, H., Tourle, S., Wahlund, E., Walden, A., Wan, J., Wernerus, H., Westberg, J., Wester, K., Wrethagen, U., Xu, L. L., Hober, S., and Ponten, F. (2005) A human protein atlas for normal and cancer tissues based on antibody proteomics. Mol. Cell Proteomics. 4, 1920-1932

4. Uhlen, M., and Ponten, F. (2005) Antibody-based proteomics for human tissue profiling. Mol. Cell Proteomics. 4, 384-393

5. Nilsson, P., Paavilainen, L., Larsson, K., Odling, J., Sundberg, M., Andersson, A. C., Kampf, C., Persson, A., Al-Khalili, S. C., Ottosson, J., Bjorling, E., Hober, S., Wernerus, H., Wester, K., Ponten, F., and Uhlen, M. (2005) Towards a human proteome atlas: high-throughput generation of mono-specific antibodies for tissue profiling. Proteomics. 5, 4327-4337

6. Martelli, P. L., Fariselli, P., and Casadio, R. (2003) An ENSEMBLE machine learning approach for the prediction of all-alpha membrane proteins. Bioinformatics. 19 Suppl 1, i205-i211

7. Pierleoni, A., Martelli, P. L., Fariselli, P., and Casadio, R. (2006) BaCelLo: a balanced subcellular localization predictor. Bioinformatics. 22, e408-e416

8. Pierleoni, A., Martelli, P.L., Casadio, R. (2011) MemLoci: predicting subcellular localization of membrane proteins in Eukaryotes. Bioinformatics, DOI: 10.1093/bioinformatics/BTR108

9. Altschul, S. F., Gish, W., Miller, W., Myers, E. W., and Lipman, D. J. (1990) Basic local alignment search tool. J. Mol. Biol. 215, 403-410

10. Aslanidis, C., and de Jong, P. J. (1990) Ligation-independent cloning of PCR products (LIC-

PCR). Nucleic Acids Res. 18, 6069-6074

11. Studier, F. W. (2005) Protein production by auto-induction in high density shaking cultures. Protein Expr. Purif. 41, 207-234

12. Kononen, J., Bubendorf, L., Kallioniemi, A., Barlund, M., Schraml, P., Leighton, S., Torhorst, J., Mihatsch, M. J., Sauter, G., and Kallioniemi, O. P. (1998) Tissue microarrays for high-throughput molecular profiling of tumor specimens. Nat. Med. 4, 844-847

13. Kampf, C., Andersson, A. C., Wester, K., Bjorling, E., Uhlen, M., and Ponten, F. (2004) Antibody-based Tissue profiling as tools for clinical proteomics, In: Clinical Proteomics, 1 Ed., pp. 285-299

14. Andreu, P., Colnot, S., Godard, C., Laurent-Puig, P., Lamarque, D., Kahn, A., Perret, C., and Romagnolo, B. (2006) Identification of the IFITM family as a new molecular marker in human colorectal tumors. Cancer Res. 66, 1949-1955

15. Cheong, S. C., Chandramouli, G. V., Saleh, A., Zain, R. B., Lau, S. H., Sivakumaren, S., Pathmanathan, R., Prime, S. S., Teo, S. H., Patel, V., and Gutkind, J. S. (2009) Gene expression in human oral squamous cell carcinoma is influenced by risk factor exposure. Oral Oncol. 45, 712-719

16.. Fan, J., Peng, Z., Zhou, C., Qiu, G., Tang, H., Sun, Y., Wang, X., Li, Q., Le, X., and Xie, K. (2008) Gene-expression profiling in Chinese patients with colon cancer by coupling experimental and bioinformatic genomewide gene-expression analyses: identification and validation of IFITM3 as a biomarker of early colon carcinogenesis. Cancer 113, 266-275

17. Kosari, F., Asmann, Y. W., Cheville, J. C., and Vasmatzis, G. (2002) Cysteine-rich secretory protein-3: a potential biomarker for prostate cancer. Cancer Epidemiol. Biomarkers Prev. 11, 1419-1426

18. Bjartell, A., Johansson, R., Bjork, T., Gadaleanu, V., Lundwall, A., Lilja, H., Kjeldsen, L., and Udby, L. (2006) Immunohistochemical detection of cysteine-rich secretory protein 3 in tissue and in serum from men with cancer or benign enlargement of the prostate gland. Prostate 66, 591-603

19. Hammarstrom, S. (1999) The carcinoembryonic antigen (CEA) family: structures, suggested functions and expression in normal and malignant tissues. Semin. Cancer Biol. 9, 67-81

20. Kuespert, K., Pils, S., and Hauck, C. R. (2006) CEACAMs: their role in physiology and pathophysiology. Curr. Opin. Cell Biol. 18, 565-571

21. Leth-Larsen, R., Lund, R. R., and Ditzel, H. J. (2010) Plasma membrane proteomics and its application in clinical cancer biomarker discovery. Mol. Cell Proteomics. 9, 1369-1382

22. Wang, Z. Q., Xing, W. M., Fan, H. H., Wang, K. S., Zhang, H. K., Wang, Q. W., Qi, J., Yang, H. M., Yang, J., Ren, Y. N., Cui, S. J., Zhang, X., Liu, F., Lin, D. H., Wang, W. H., Hoffmann, M. K., and Han, Z. G. (2009) The novel lipopolysaccharide-binding protein CRISPLD2 is a critical serum protein to regulate endotoxin function. J. Immunol. 183, 6646-6656

23. Wu, F., Dassopoulos, T., Cope, L., Maitra, A., Brant, S. R., Harris, M. L., Bayless, T. M., Parmigiani, G., and Chakravarti, S. (2007) Genome-wide gene expression differences in Crohn's disease and ulcerative colitis from endoscopic pinch biopsies: insights into distinctive pathogenesis. Inflamm. Bowel. Dis. 13, 807-821

24.. Hisamatsu, T., Watanabe, M., Ogata, H., Ezaki, T., Hozawa, S., Ishii, H., Kanai, T., and Hibi, T. (1999) Interferon-inducible gene family 1-8U expression in colitis-associated colon cancer and severely inflamed mucosa in ulcerative colitis. Cancer Res. 59, 5927-5931

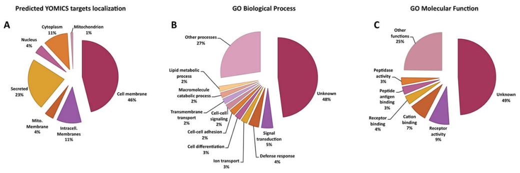
Predicted YOMICS targets localization
A
Cytoplasm
11%
Mitochondrion
1%
Nucleus
4%
Secreted
23%
Mito.
Membrane
4%
Intracell.
Membranes
11%
Cell membrane
46%
GO Biological Process
B
Other processes
27%
Lipid metabolic
process
2%
Macromolecule
catabolic process
2%
Transmembrane
transport
2%
Cell-cell
signaling
2%
Cell-cell adhesion
2%
Cell differentiation
3%
Ion transport
3%
Defense response
4%
Signal
transduction
5%
Unknown
48%
GO Molecular Function
C
Other
functions
25%
Peptidase
activity
3%
Peptide
antigen
binding
3%
Receptor
binding
4%
Cation
binding
7%
Receptor
activity
9%
Unknown
49%

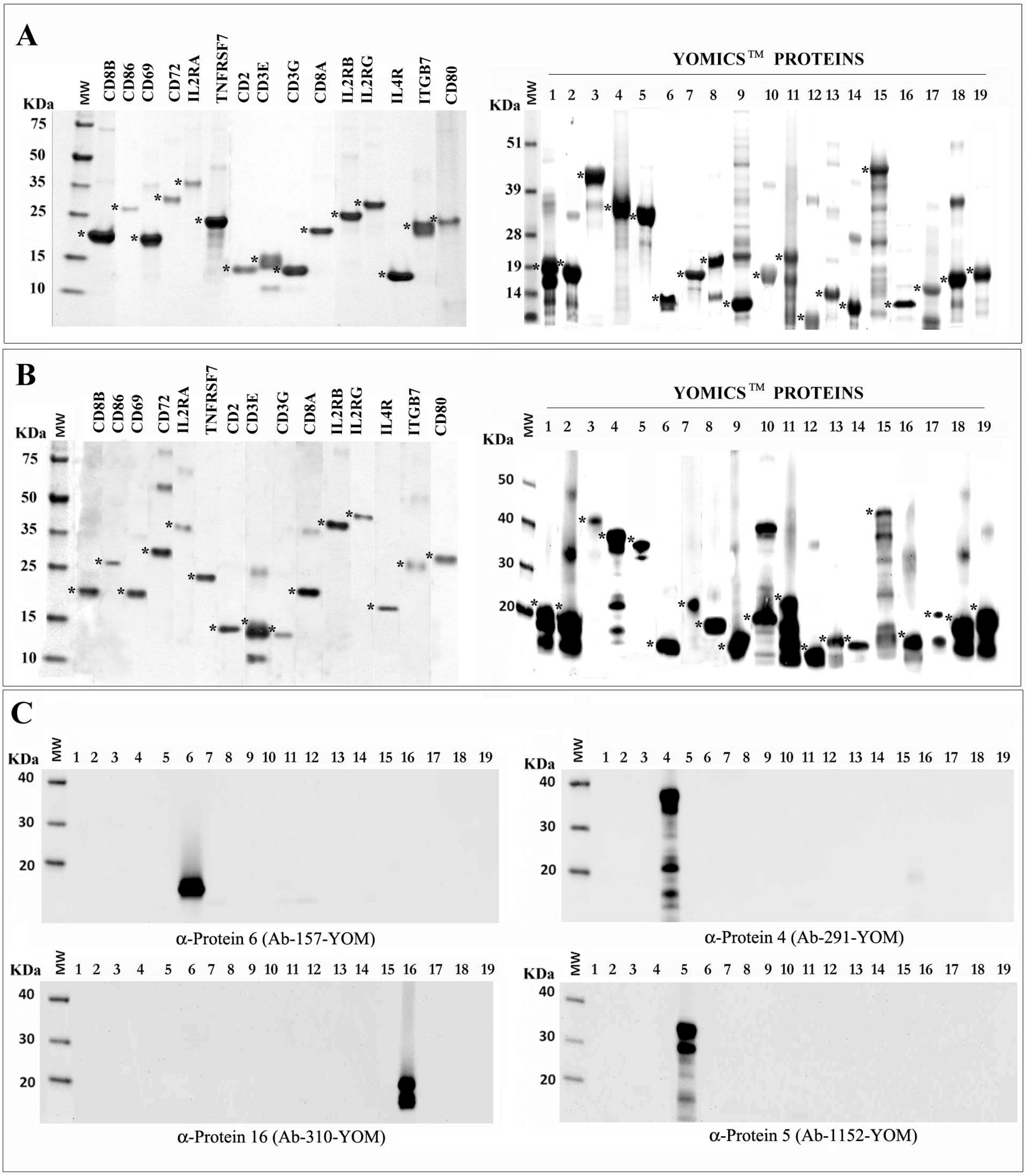
A
KDa
MW
CD8B
CD86
CD69
CD72
IL2RA
TNFRSF7
CD2
CD3E
CD3G
CD8A
IL2RB
IL2RG
IL4R
ITGB7
CD80
75
50
35
25
15
10
YOMICS™ PROTEINS
1 2 3 4 5 6 7 8 9 10 11 12 13 14 15 16 17 18 19
51
39
28
19
14
B
KDa
MW
CD8B
CD86
CD69
CD72
IL2RA
TNFRSF7
CD2
CD3E
CD3G
CD8A
IL2RB
IL2RG
IL4R
ITGB7
CD80
75
50
35
25
15
10
YOMICS™ PROTEINS
1 2 3 4 5 6 7 8 9 10 11 12 13 14 15 16 17 18 19
50
40
30
20
C
KDa
MW
1 2 3 4 5 6 7 8 9 10 11 12 13 14 15 16 17 18 19
40
30
20
α-Protein 6 (Ab-157-YOM)
α-Protein 4 (Ab-291-YOM)
α-Protein 16 (Ab-310-YOM)
α-Protein 5 (Ab-1152-YOM)

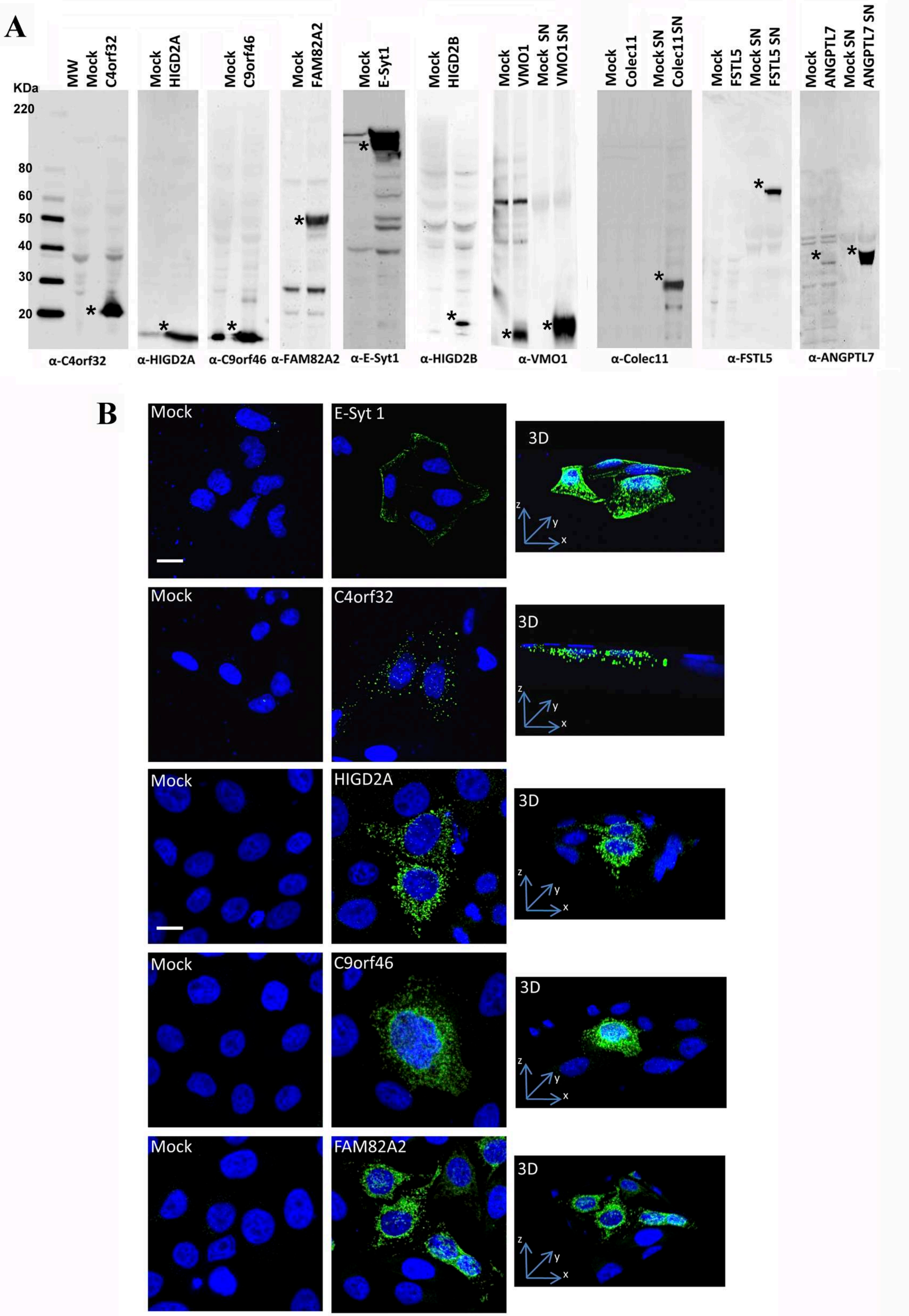
A
KDa
220
80
60
50
40
30
20
MW
Mock
C4orf32
Mock
HIGD2A
Mock
C9orf46
Mock
FAM82A2
Mock
E-Syt1
Mock
HIGD2B
Mock
VMO1
Mock SN
VMO1 SN
Mock
Colec11
Mock SN
Colec11 SN
Mock
FSTL5
Mock SN
FSTL5 SN
Mock
ANGPTL7
Mock SN
ANGPTL7 SN
α-C4orf32
α-HIGD2A
α-C9orf46
α-FAM82A2
α-E-Syt1
α-HIGD2B
α-VMO1
α-Colec11
α-FSTL5
α-ANGPTL7
B
Mock
E-Syt 1
3D
Mock
C4orf32
3D
Mock
HIGD2A
3D
Mock
C9orf46
3D
Mock
FAM82A2
3D
z
y
x

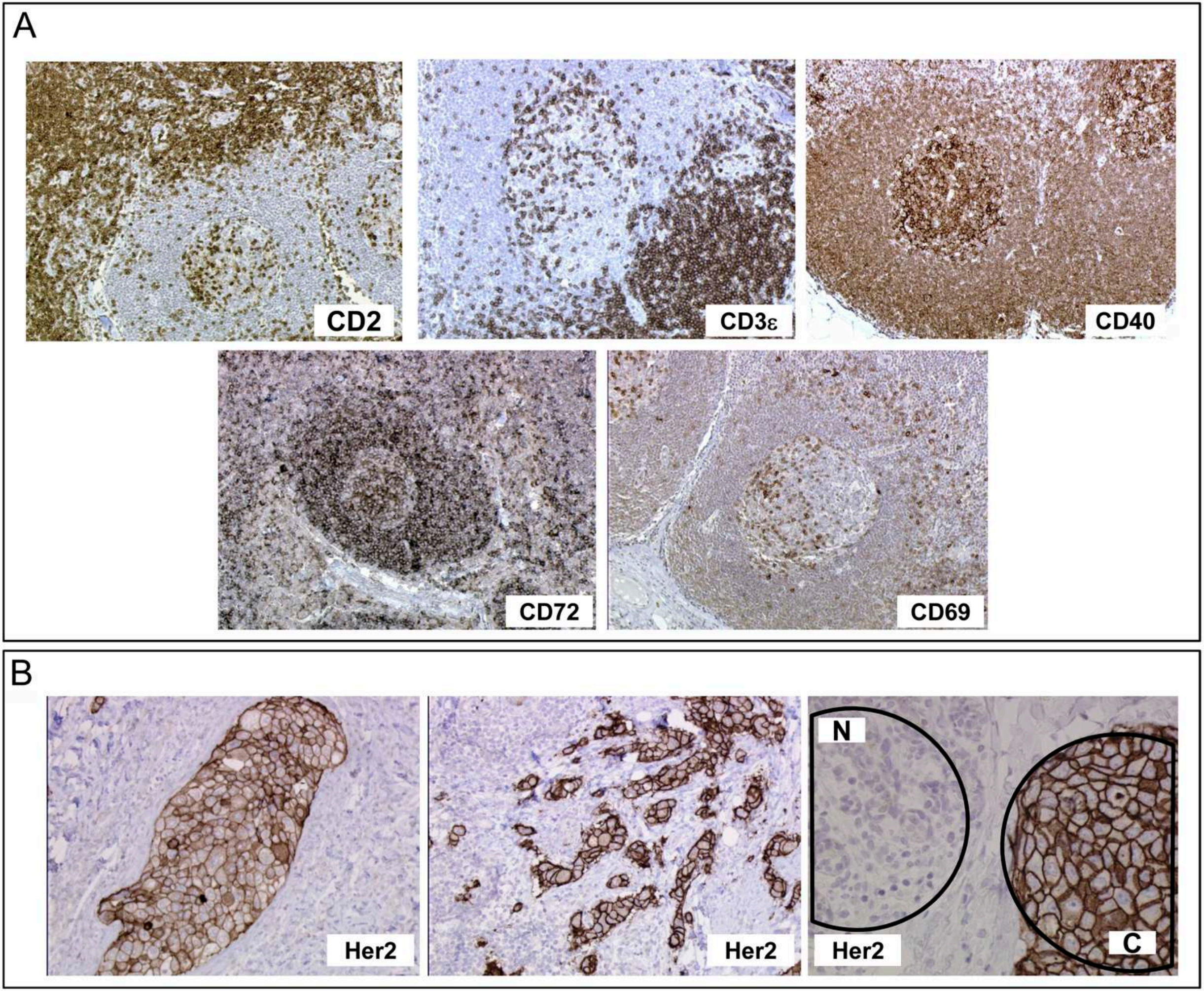
A
CD2
CD3ε
CD40
CD72
CD69
B
Her2
Her2
N
Her2
C

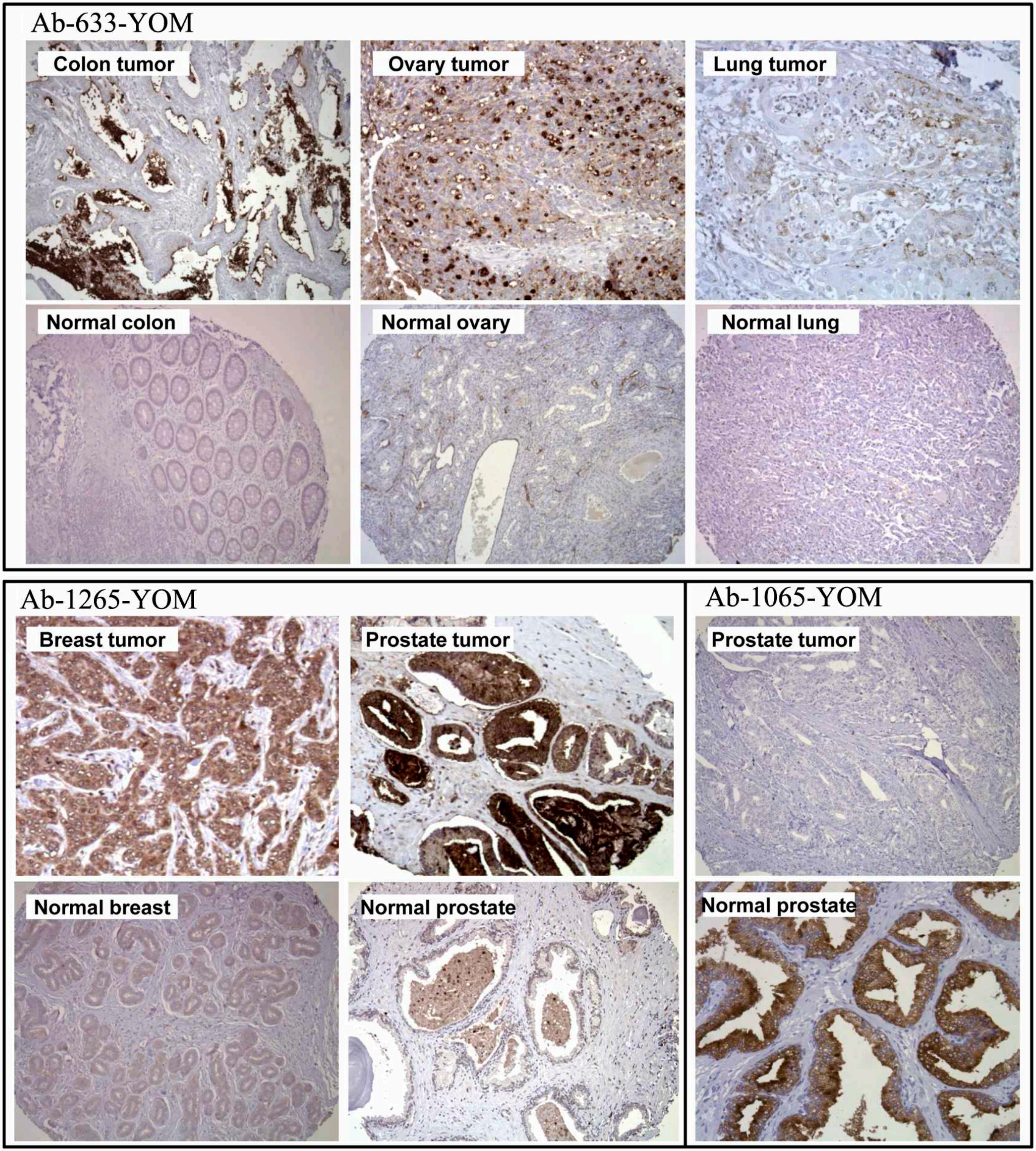
Ab-633-YOM
Colon tumor
Ovary tumor
Lung tumor
Normal colon
Normal ovary
Normal lung
Ab-1265-YOM
Breast tumor
Prostate tumor
Normal breast
Normal prostate
Ab-1065-YOM
Prostate tumor
Normal prostate

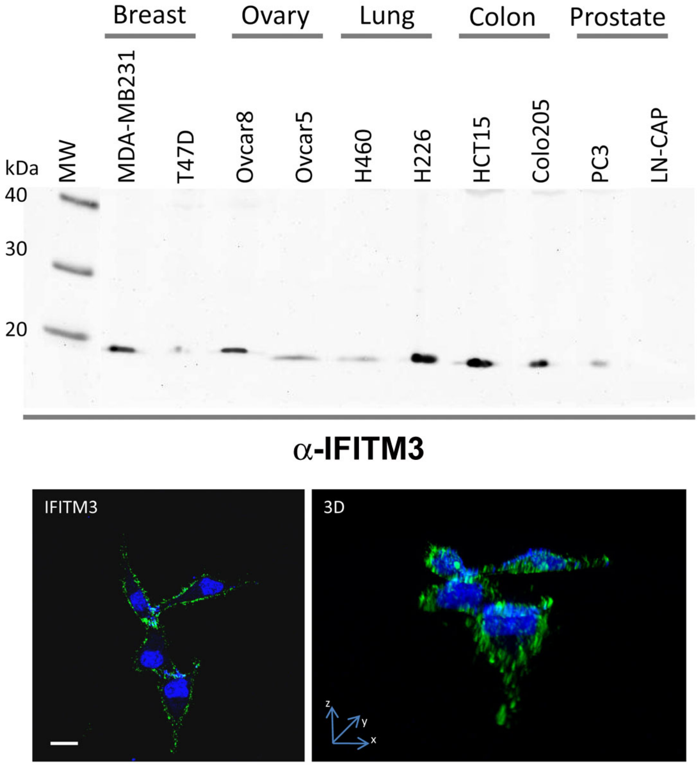
Breast
Ovary
Lung
Colon
Prostate
kDa
MW
MDA-MB231
T47D
Ovcar8
Ovcar5
H460
H226
HCT15
Colo205
PC3
LN-CAP
40
30
20
α-IFITM3
IFITM3
3D
z
y
x
