# Supplementary figures and images for: Identification of New Hematopoietic Cell Subsets with a Polyclonal Antibody Library Specific for Neglected Proteins
Source: PLoS One. 2012 Apr 4;7(4):e34395. doi: 10.1371/journal.pone.0034395 (PMC3319577; doi:10.1371/journal.pone.0034395)

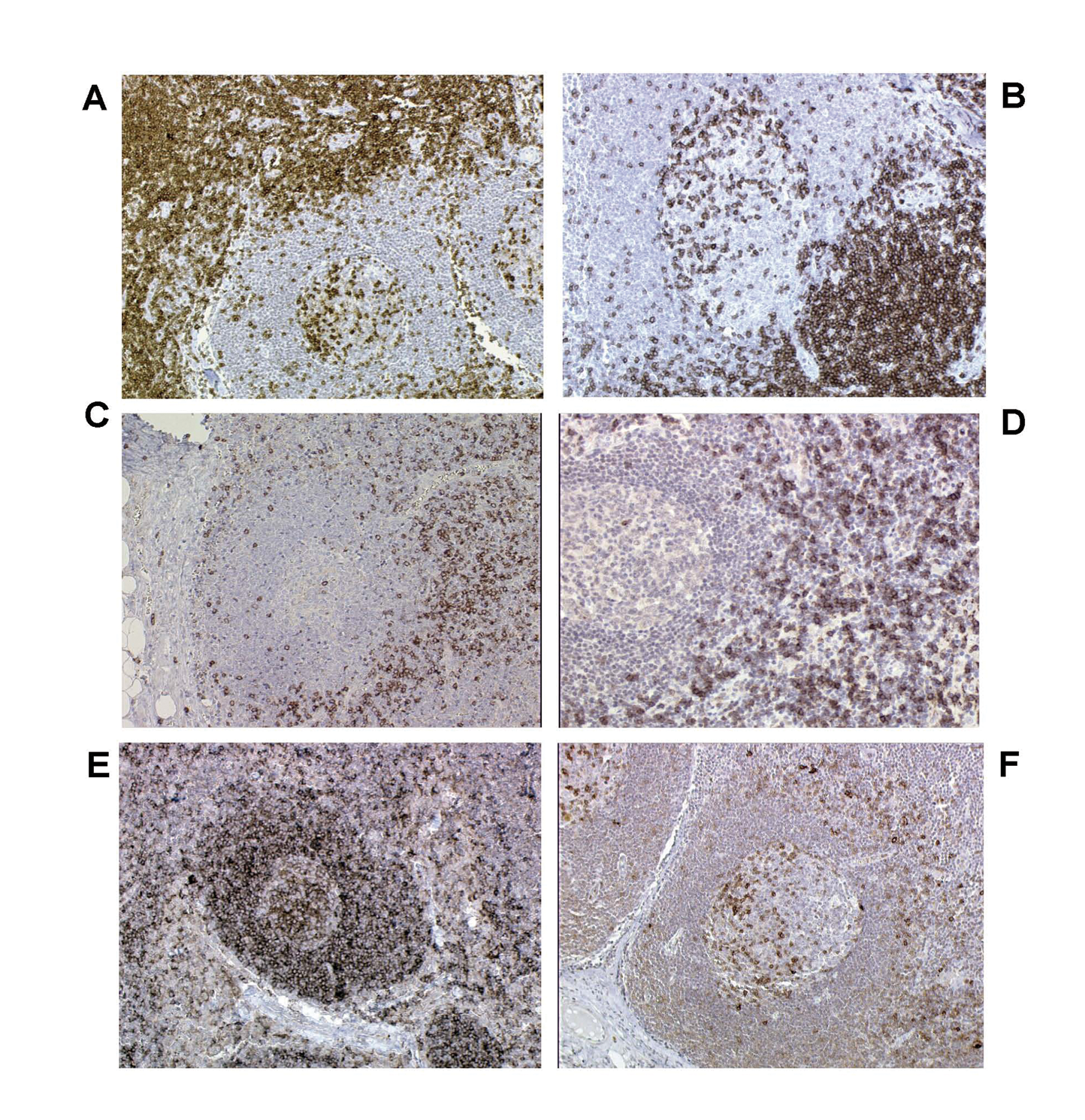

Supplement: Figure S1 — IHC analysis with sera specific for well-known proteins. Sections of human lymph nodes were pretreated with an antigen retrieval solution and were then incubated with the indicated antisera. Detection steps were done using a commercially available kit according to the manufacturer instructions. Peroxidase activity was developed with 3-3-diaminobenzidine-copper sulfate to obtain a brown-black end product. A) anti CD2, B) anti CD3 gamma, C) anti CD8 alpha, D) anti CD8 beta, E) anti CD72, F) anti CD69. (TIFF) [file pone.0034395.s002.tif]

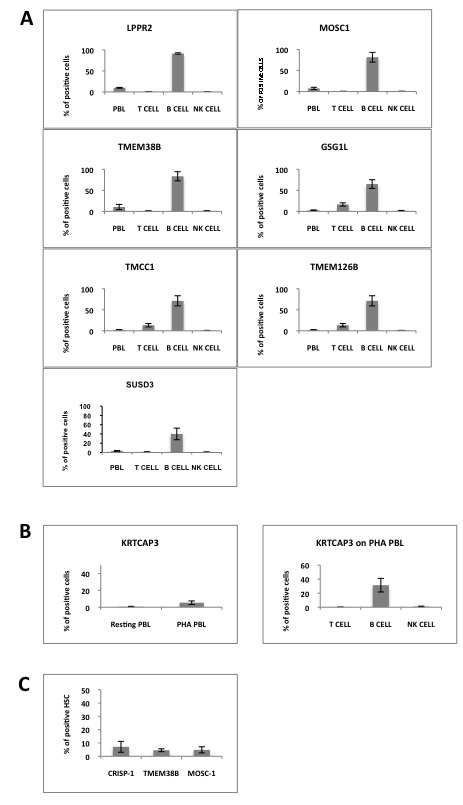

Supplement: Figure S2 — Results of sera screening by FACS on PBLs and cord blood cells. A) FACS analysis of sera positive on PBLs. PBLs were stained with the indicated sera. The samples were also stained with anti CD3, anti CD19 and anti CD56 mAbs to analyze the sera reactivity upon gating on the different subpopulations. B) PBMCs are treated for 24 hours with 1 µg/ml of PHA. After the treatment both un-stimulated and stimulated cells are stained with KRTCAP-3-specific serum. C) FACS analysis of sera positive on cord blood cells. Cord blood mononuclear cells are stained with the indicated sera. The samples are stained also with anti CD45 and anti CD34 mAbs to perform the analysis upon gating on CD34high CD45dim cells. In all the cases the average with the relative standard deviation of five different donors is shown for each serum. (TIFF) [file pone.0034395.s003.tif]

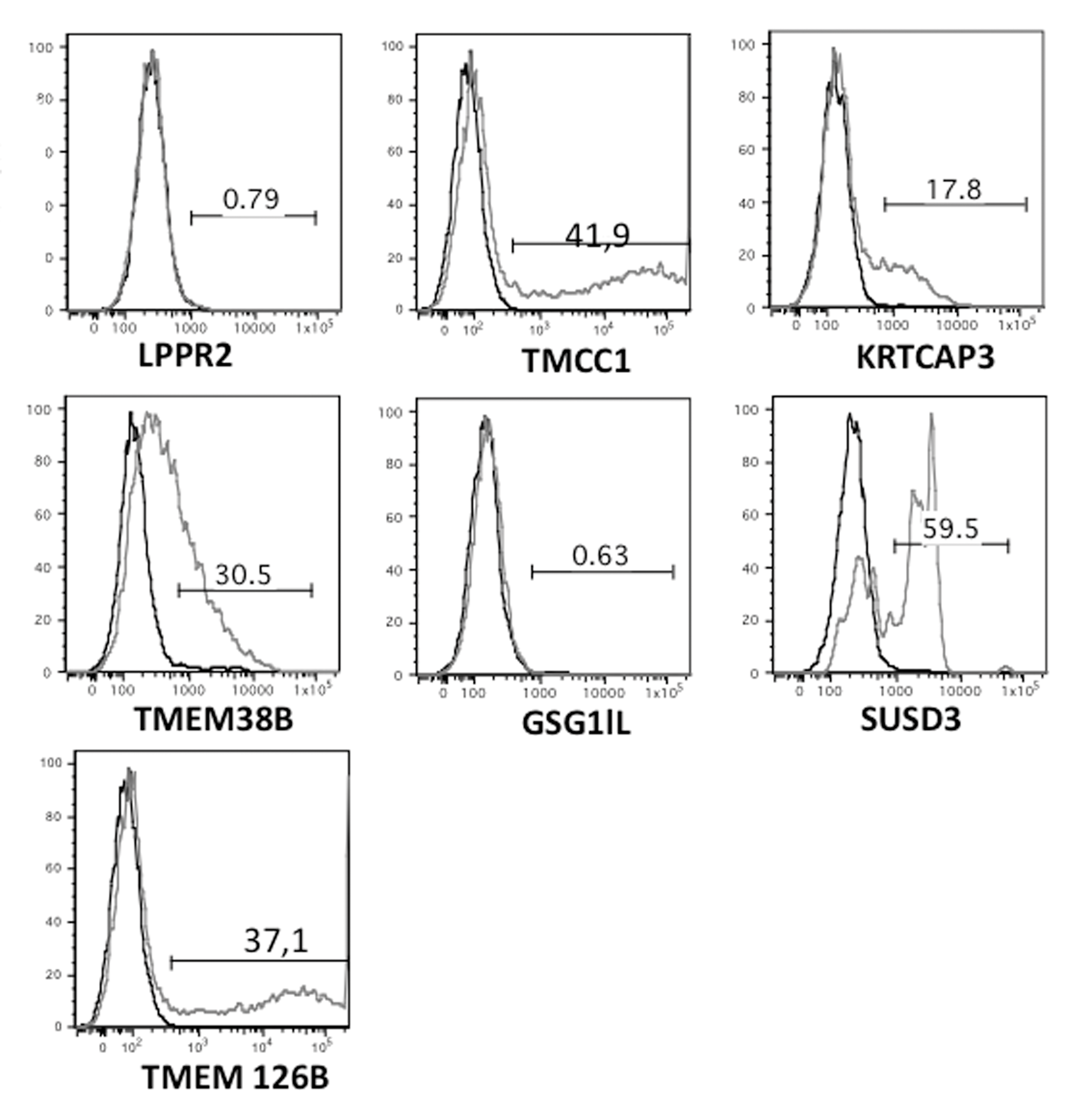

Supplement: Figure S3 — Assessment of antisera specificity on Hela transfected cells by FACS analysis. Hela cells were transfected with a myc-tag version of the proteins identified with the sera library. At 24 hours from the transfection cells were stained with the indicated sera as described in Methods section. As negative control un-transfected wt Hela cells stained with the same antisera were used. The FACS analysis for the CRISP-1 and MOSC-1 proteins is not shown since the two proteins are secreted and mouse antisera cannot be used in intra cellular staining because of the high background. (TIFF) [file pone.0034395.s004.tif]

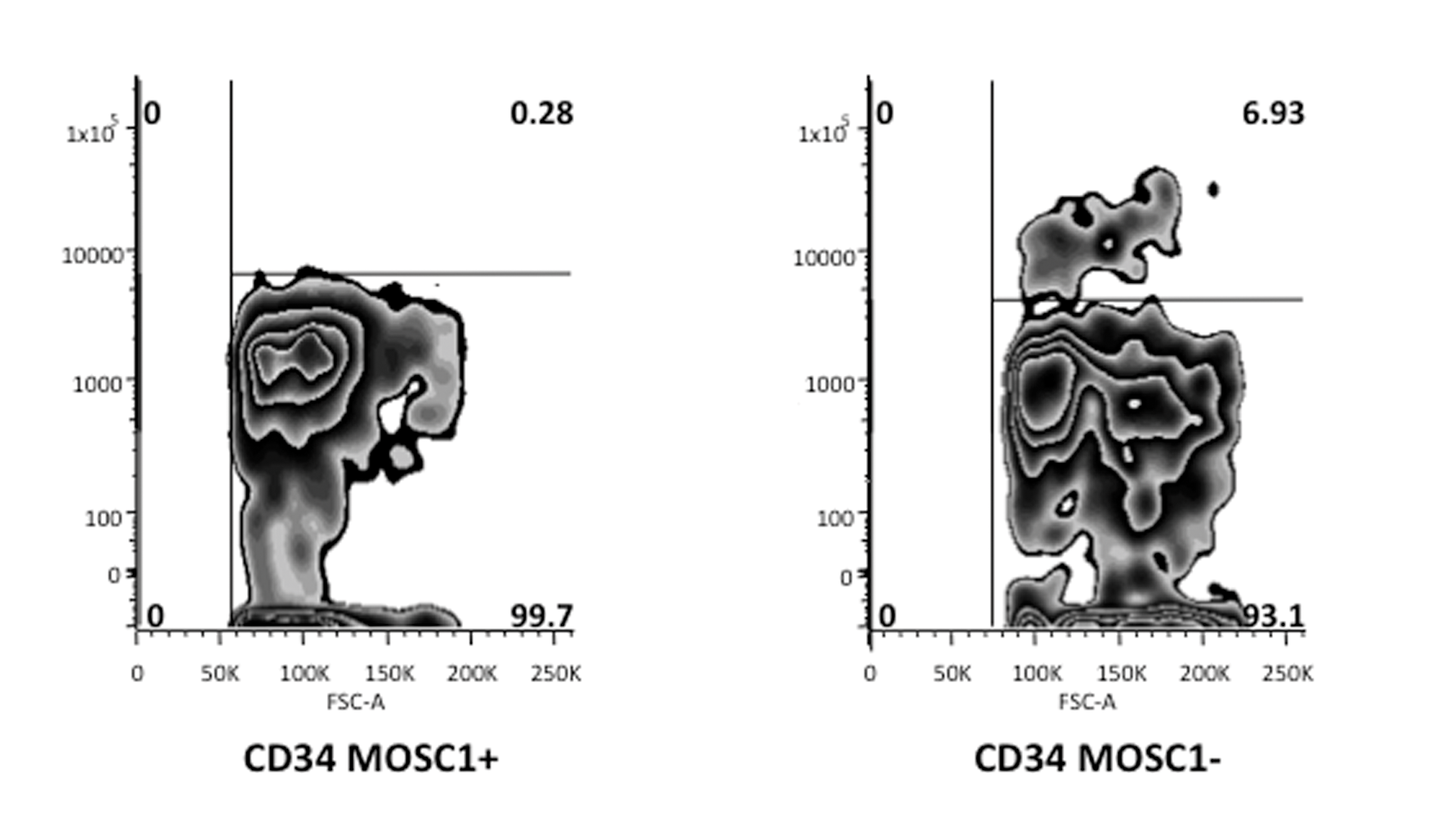

Supplement: Figure S4 — Differentiation assay on OP9 cells. Because of the reactivity of MOSC-1-specific antiserum on B cells, we have tested the capability of CD34+ cells expressing MOSC-1 to generate mature B cells upon differentiation on OP9 stromal cells. MOSC-1 positive and negative cells were sorted by FACS (purity >98%) and analyzed for the up-regulation of the B cell marker CD19 upon 14 days of culture on OP9 cells. 500 cells were plated for each condition. MOSC-1 positive cells were unable to generate B cells in this assay. This result is in agreement with the hypothesis that MOSC-1 is expressed only by mature monocytes and binds a receptor on B cells. (TIFF) [file pone.0034395.s005.tif]
